# Drug resistance of *Plasmodium falciparum* and *Plasmodium vivax* isolates in Indonesia

**DOI:** 10.1186/s12936-022-04385-2

**Published:** 2022-11-28

**Authors:** Farindira Vesti Rahmasari, Puji B. S. Asih, Farahana K. Dewayanti, Chawarat Rotejanaprasert, Prakaykaew Charunwatthana, Mallika Imwong, Din Syafruddin

**Affiliations:** 1 Graduate Program in Molecular Medicine, Faculty of Science, Mahidol University, Bangkok, 10400 Thailand; 2 Department of Molecular Tropical Medicine and Genetics, Faculty of Tropical Medicine, Mahidol University, Bangkok, Thailand; 3 Department of Parasitology, School of Medicine, Faculty of Medicine and Health Sciences, Universitas Muhammadiyah Yogyakarta, Yogyakarta, Indonesia; 4 Eijkman Research Centre for Molecular Biology, National Research and Innovation Agency, Jakarta, Indonesia; 5 Mahidol-Oxford Tropical Medicine Research Unit (MORU), Faculty of Tropical Medicine, Mahidol University, Bangkok, Thailand; 6 Department of Clinical Tropical Medicine, Faculty of Tropical Medicine, Mahidol University, Bangkok, Thailand; 7 Department of Parasitology, Faculty of Medicine, Hasanuddin University, Makassar, Indonesia; 8 Department of Tropical Hygiene, Faculty of Tropical Medicine, Mahidol University, Bangkok, Thailand

**Keywords:** *P. falciparum*, *P. vivax*, Indonesia, Molecular, Drug resistance

## Abstract

This review article aims to investigate the genotypic profiles of *Plasmodium falciparum* and *Plasmodium vivax* isolates collected across a wide geographic region and their association with resistance to anti-malarial drugs used in Indonesia. A systematic review was conducted between 1991 and date. Search engines, such as PubMed, Science Direct, and Google Scholar, were used for articles published in English and Indonesian to search the literature. Of the 471 initially identified studies, 61 were selected for 4316 *P. falciparum* and 1950 *P. vivax* individual infections. The studies included 23 molecular studies and 38 therapeutic efficacy studies. K76T was the most common *pfcrt* mutation. K76**N** (2.1%) was associated with the haplotype CVMN**N**. By following dihydroartemisinin–piperaquine (DHA–PPQ) therapy, the mutant *pfmdr1* alleles 86Y and 1034C were selected. Low prevalence of haplotype N86**Y**/Y184/D1246**Y** *pfmdr1* reduces susceptibility to AS–AQ. SNP mutation *pvmdr1* Y976F reached 96.1% in Papua and East Nusa Tenggara. Polymorphism analysis in the *pfdhfr* gene revealed 94/111 (84.7%) double mutants S108**N**/C59R or S108**T**/A16**V** in Central Java. The predominant *pfdhfr* haplotypes (based on alleles 16, 51, 59,108, 164) found in Indonesia were ANC**N**I, ANCSI, AN**RN**I, and AN**RN**L. Some isolates carried A437**G** (35.3%) or A437**G**/K540**E** SNPs (26.5%) in *pfdhps*. Two novel *pfdhps* mutant alleles, I588**F**/**G** and K540**T**, were associated with six *pfdhps* haplotypes. The highest prevalence of *pvdhfr* quadruple mutation (F57**L/**S58**R/**T61**M**/S117**T**) (61.8%) was detected in Papua. In *pvdhps*, the only polymorphism before and after 2008 was 383G mutation with 19% prevalence. There were no mutations in the *pfk13* gene reported with validated and candidate or associated *k13* mutation. An increased copy number of *pfpm2*, associated with piperaquine resistance, was found only in cases of reinfection. Meanwhile, mutation of *pvk12* and *pvpm4* I165V is unlikely associated with ART and PPQ drug resistance. DHA–PPQ is still effective in treating uncomplicated falciparum and vivax malaria. Serious consideration should be given to interrupt local malaria transmission and dynamic patterns of resistance to anti-malarial drugs to modify chemotherapeutic policy treatment strategies. The presence of several changes in *pfk13* in the parasite population is of concern and highlights the importance of further evaluation of parasitic ART susceptibility in Indonesia.

**Graphical Abstract:**

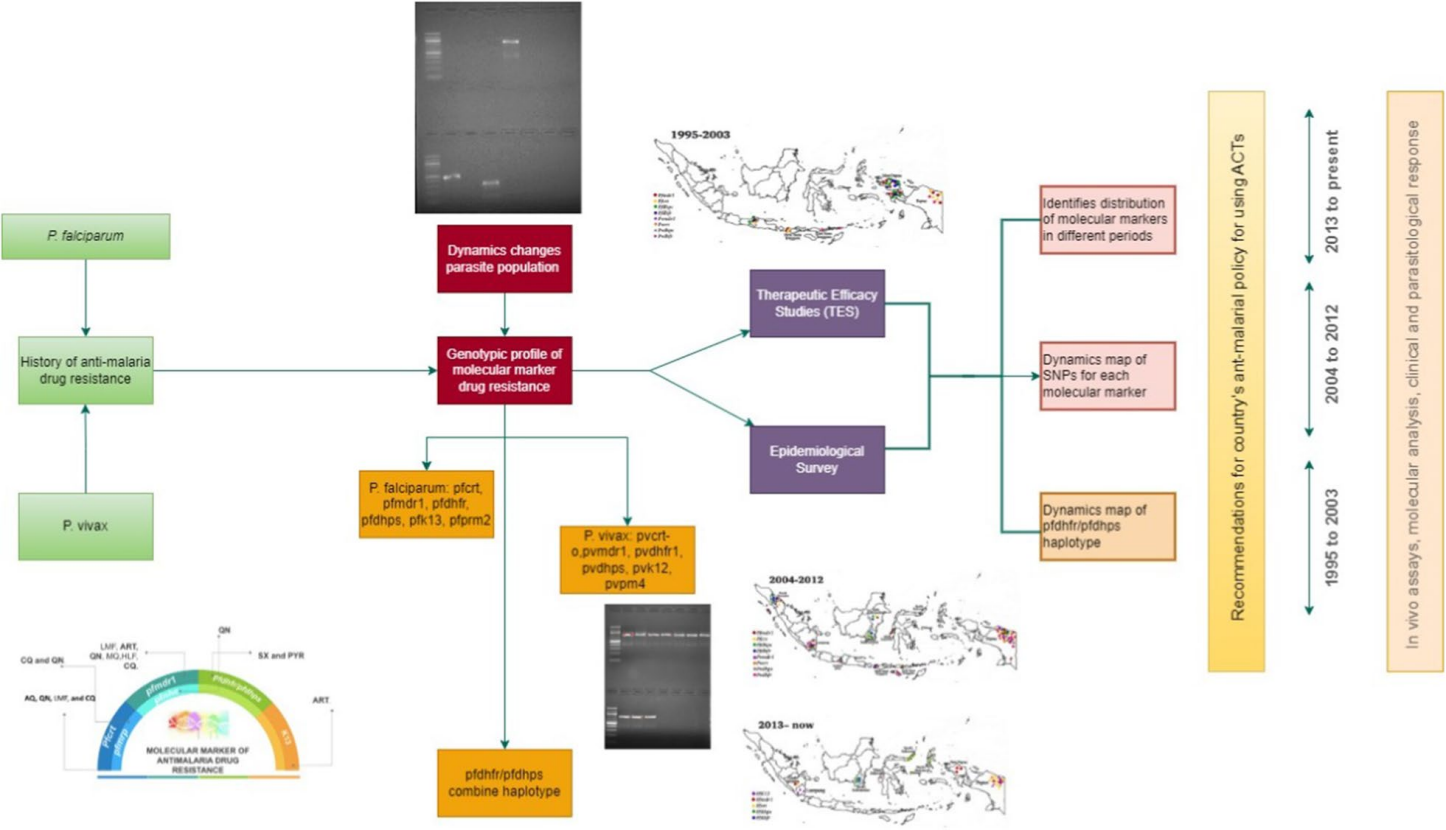

**Supplementary Information:**

The online version contains supplementary material available at 10.1186/s12936-022-04385-2.

## Background

Malaria is an infectious disease that still poses a public health problem in 87 countries worldwide. In 2020, 1 year after the COVID-19 pandemic and service disruptions, the number of malaria cases rose to 241 million, an additional 14 million cases compared with 2019. In 2020, malaria deaths increased by 12% compared with 2019, to 627,000, an estimated additional 69,000 deaths. Nine countries in the World Health Organization (WHO) Southeast Asia Region were endemic for malaria in 2020, with approximately 5 million cases accounting for 2% of the global malaria caseload. Despite significant progress in reducing malaria incidence, Indonesia remains one of Southeast Asia’s nine malaria-endemic countries. It is responsible for 21% of reported cases and 6% of deaths in the area [1–3].

The rapid emergence and spread of parasite strains resistant to anti-malarial drug mainstay, such as chloroquine (CQ), sulfadoxine–pyrimethamine (SP), and, more recently, artemisinin (ART)-based combination therapy (ACT) poses a constant challenge to the malaria control and elimination programmes. It proposes to eliminate malaria in all provinces in Indonesia by 2030 based on regional target, with Jawa-Bali in 2023, Sumatra, Sulawesi, West Nusa Tenggara in 2025, Kalimantan and North Maluku in 2027, Maluku and East Nusa Tenggara in 2028 and Papua, West Papua in 2029 [3, 4]. Indonesia's malaria control and elimination programmes have successfully eliminated malaria in 67% of the 514 regencies and municipalities. However, malaria is still persistently highly endemic in Papua, West Papua, and East Nusa Tenggara provinces. It represents the vast majority of the country’s cases [5, 6]. Since 2004, Indonesia adopted ACT as the first-line anti-malarial drug regimen to replace the failing CQ and SP. However, to prevent ACT resistance, the government has tightened controls on ACT deployment. Only those with a proven laboratory diagnosis, either by microscopy or rapid diagnostic test, will receive DHA–PPQ [7].

In addition, anti-malarial drugs are divided into different families, some of which are still in use or removed due to resistance [8–15]. The history of using anti-malarial drugs, as described in Table 1, provides a detailed description of the development of anti-malarial drugs in several regions in Indonesia. Quinolines are the oldest type of anti-malarial medication. Quinine (QN) was the first medicine in this class to be proven safe and effective, and it is still used as a second-line treatment for severe malaria to this day for both *Plasmodium* sp. [16]. Since the first reports of CQ resistance in East Kalimantan and Indonesian Papua Provinces in 1975, CQ resistance has been documented in all parts of the archipelago through in vivo and in vitro drug tests [8–15]. except in one remote area in Indonesia, CQ therapy is more amenable to treatment and control [15].

**Table 1:** History of anti-malarial drug use in several areas in Indonesia

Research title/source	Sites	Year						
		SP	DHA–PPQ	PQ	QN	AL	AS–AQ	CQ
[151]	Purworejo District, Central Java Province	Before 2004	2004–now	2004–now	–	–	–	Before 2004
[152]	Southwest Sumba District, East Nusa Tenggara Province	1986–2005	2005–now	2005–now	All years before 1986–now	Until 2005	Until 2005	1986–2008
[152]	West Nusa Tenggara Province	1986–March 2008	April 2008–now	2008–now	All years before 1986–now	–	Until 2008	1986–2008
[153]	Kulon Progo, Yogyakarta Province, Java Island	2000–2005	2005–now	1998–now	–	–	–	1997–2000
[154]	Gunung Kidul, Yogyakarta Province, Java Island	–	After 2014	After 2014	Before 2014	–	–	Before 2014
[155]	Wonosobo District, Central Java Province	1997–2005	2007 until now	–	–	–	Before 2007	1997–2005
[156]	Magelang District, Central Java Province	–	2007 until now	–	–	–	Before 2007	Before 2007
[157]	Cilacap District, Central Java Province	Before 2005	2005–until now	2005–now	–	–	–	Before 2005
[158]	Jepara District, Central Java Province	Before 2005	2005–now	2005–now	–	–	–	Before 2005
[159]	Lampung Province, Sumatra Island	Until 2008	2012–now	2004–now	–	–	Until 2008	Until 2008
[160]	Gorontalo District, Sulawesi Province	–	2012–now	2012–now	All years	–	2010–2012	Before 2010
[161]	Makassar, Sulawesi Province	–	2010–now	–	–		–	Before 2010
[162]	Sulawesi Province	Before 2010	2016–now	–	–	–	2010–2012	Before 2010
[35]	Timika, Papua Province	Before 2006	2006–now	–	–	–	Until–2006	Before 2006

Reports of decreasing CQ efficacy have highlighted the necessity for alternative *P. falciparum* and *P. vivax* treatment options [15, 17]. Furthermore, molecular studies have provided evidence that polymorphisms in the *P. falciparum chloroquine resistance transporter (pfcrt)* and *P. falciparum multidrug resistance 1 (pfmdr1)* genes modulate higher levels of CQ, mefloquine (MQ), halofantrine (HL), and QN resistance [18–22]. Due to increased cases of CQ treatment failure in various parts of Indonesia [23–25], a combination of antifolates, pyrimethamine (PYR), and sulfadoxine (SX) have become the first-line drug [26, 27]. This combination uses inhibitors of dihydrofolate reductase (DHFR) and dihydropteroate synthase (DHPS) [28]. The molecular basis of resistance to PYR and SX has been more clearly defined. From 1996 until 2015, several Indonesian studies in Indonesia have revealed many molecular markers in several codons responsible for antifolates resistance. SNPs mutation in *pfdhfr* such as A16**V**, C59**R**, S108**R/N/T**, I164**L**, and N51**I** were associated with PYR resistance. Meanwhile, SNPs mutation in *pfdhp*s such as A437**G**, K540**E**, A581**T**, I588**G**, and I588**F** were linked to SX resistance [29–36]. The failure of SP was first reported in 1979 [26, 27, 37–39]. However, resistance to this medication combination was discovered in Indonesia and has spread throughout the archipelago [13, 40–43].

The global deployment of ACT to treat asexual blood-stage infections was recommended by the WHO and has successfully decreased the global prevalence of malaria [44, 45]. The Ministry of Health (MoH) Republic of Indonesia adopted ACT in its treatment strategy in 2004. To avoid the drug resistance problem, the National Malaria Control Programme, Republic of Indonesia, has taken steps to ensure effective drug administration and regulation [7, 16, 46]. ACT is an anti-malarial drug regimen that combines ART derivatives with other anti-malarial drugs, including lumefantrine (LUM), amodiaquine (AQ), MQ, piperaquine (PPQ), and SP [47]. Initially, the MoH used artesunate–amodiaquine (AS–AQ) as the first-line drug. However, several therapeutic efficacy studies reported high failure of these combinations in Central Java, Papua, and Sumatra [48, 49]. AS–AQ has lower efficacy than AL for treating uncomplicated malaria in children [50]. In line with a study from Hasugian et al. comparing two artemisinin-based combinations, patients treated with AS–AQ have a higher risk of failure than those treated with DHA–PPQ. The authors concluded that DHA–PPQ was a better tolerated and more efficient treatment for the multidrug-resistant *P. vivax* in Papua [49]. An artemisinin-based combination, dihydroartemisinin–piperaquine (DHA–PPQ), was recommended as a first-line treatment for falciparum and vivax malaria in 2008 [7]. Price et al. evaluated DHA–PPQ for *P. vivax* infection between 2004 and 2005. The study showed that the median time to recurrence was 43 days (range 22–45 days), and DHA–PPQ was an effective treatment of *P. vivax* in Papua [49]. In addition, for *P. vivax* therapy, Primaquine (PQ) will be given for 14 days with the dosage of 0.25 mg/BW/day. If it relapses, an additional dosage will be necessary (0.5 mg/BW/day) [7]. Later, in 2012, DHA–PPQ was adopted as the only ACT used for uncomplicated malaria cases throughout Indonesia. ART and its derivatives rapidly clear parasite load in the blood, within a few hours after oral administration, and yield a decrease in gametocyte carriage. The parasites are normally cleared after three days and the partner drug, which has a longer plasma half-life, is responsible for eliminating any surviving parasites [51, 52]. Therefore, successful treatment with ACT may depend on the parasite’s response to the partner drug, transmission severity, and parasite load [52, 53]. The combination of DHA–PPQ has contributed to new results of the piperaquine resistance marker, which is the copy number of *plasmepsin* 2–3. Patients with multicopy-*plasmepsin2* parasites were 20 times more likely to experience treatment failure [53–55]. Although ART has been a component of ACT, the drug has been used as monotherapy in many Greater Mekong Subregion communities. As evidenced by a delay in parasite clearance, the situation has rapidly selected for *P. falciparum* and *P. vivax* resistance to ART in the region [56–60]. To monitor the efficacy of the DHA–PPQ, the MoH regularly conducted therapeutic efficacy studies (TES) [61].

Despite a long history since anti-malarial drug resistance emerged in the 1970s with several studies revealing the molecular basis of drug resistance in *P. falciparum* and *P. vivax* isolates, no systematic review reported the genotypic profile of molecular maker drug resistance based on TES study or epidemiological survey of malaria in Indonesia has yet been undertaken. Genotyping has been proposed to identify early dynamics changes in the parasite population and genetic diversity [42, 62, 63].

This study reviewed all publications on anti-malarial drug resistance in Indonesia since 1991. In addition, the frequency distribution of SNPs of relevant gene(s) related to resistance to anti-malarial drug mainstay, CQ, SP, and ACT was included. Importantly, molecular drug resistance studies indicated that the time and spatial distribution of malaria cases reflect an epidemiological process. Although broad in scope, this review highlights the need to understand the dynamics pattern of anti-malarial drug resistance in different periods. First, the authors describe the history of anti-malarial drug use in several areas in Indonesia. Second, it identifies information about the distribution of molecular markers associated with drug resistance in different periods. Third, dynamics map of SNPs prevalence mutant allele for each molecular marker associated with drug resistance based on spatial and temporal in three time periods 1995–2003, 2004–2012, and 2013–present of *P. falciparum* and *P. vivax* isolates include putative mutations in Indonesia. Finally, this review provides the following data that should be beneficial to prevent local malaria transmission and treatment strategy development as the Indonesian government recommends modifying chemotherapeutic treatment plans that effectively prevent further development of the resistance and mitigate or eliminate malaria transmission in the country. It can help guide the country’s anti-malarial policy for using ACT.

## Methods

### Study identification

The review was carried out following a predefined protocol and described as per Preferred Reporting Items for Systematic Review and Meta-Analysis (PRISMA) recommendations [42]. A computerized search was carried out with references that were screened using literature descriptors for PubMed as follows: (over 20 years, ending March 2022) combining the terms [(falciparum OR vivax resistance, Indonesia)] AND with different combinations of ACT (chloroquine) OR (quinine OR kina, which is the local Indonesia term of tree for quinine, *Cinchona* spp.) which has been known to humans since ancient times was first discovered by the Peru Indians as a cure for malaria, because this plant contains quinoline alkaloids (quinoline) on the bark, which OR quinidine OR amodiaquine OR mefloquine OR lumefantrine OR halofantrine OR dihydroartemisinin OR dihydroartemisinin–piperaquine OR artemisinin OR artemether OR artesunate OR pyrimethamine sulphadoxine OR trimethoprim OR pyrimethamine OR sulphadoxine OR antifolates OR primaquine OR artemether OR artemether lumefantrine AND according to single-nucleotide polymorphisms known to be associated with treatment failure (mutation OR polymorphism OR SNP OR mutant allele OR mutant or allele change OR allelic changing). PubMed, Science Direct, and Google Scholar databases implemented the complete search strategies.

### Study selection

Four authors (FVR, PBSA, FKD, and CR) independently reviewed abstracts and the full text of the references identified to select the articles for inclusion suitability. They extracted the data, with disagreements addressed and agreement with other authors (MI, DS, and PC). The authors considered studies of various designs that identified molecular profiles of anti-malarial drug resistance across a wide geographic region of Indonesia in *P. falciparum* and *P. vivax* isolates. However, examiners were not blinded to authors, institutions, or journal names.

### Inclusion criteria

Studies would be included in the analysis if all of the following distinguishing features could be obtained from the publication:

1. Published in English and Indonesian.

2. Original articles and short reports, but no review articles.
3. Patients with uncomplicated *P. falciparum* or *P. vivax* infections in Indonesia since 1991.
4. The standards of drug resistance were defined following the WHO guidelines.
5. A study comprising an analysis of anti-malarial drug resistance molecular marker.

### Data extraction

**Fig. 1:**
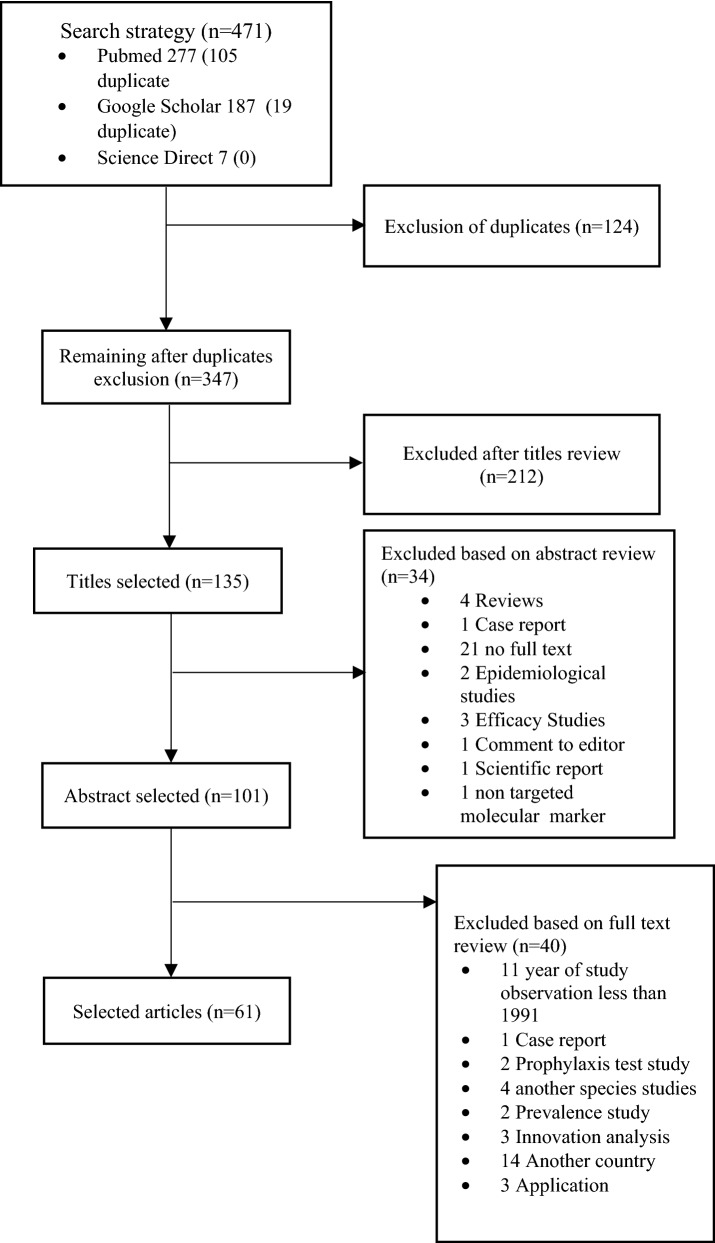
Search strategy. Forty studies have been omitted after the application of selection criteria

Data were extracted using a standard form created particularly for this review. The sections entered in the form were as follows: study identification (study title, author, journal, year of collecting sample, year of publication, country, language, and financial institution), study characteristics (design, number of patients, number of sites, and study period), and population characteristics under study (drug type, hemoglobin, gametocytes, days of follow-up, gender, therapeutic response, *P. falciparum* count, molecular marker, SNPs, SNPs frequency). Therefore, this systematic review focuses on reporting studies in evidence, their findings, and qualitative synthesis. Figure 1 shows the search strategy used. The list of 61 articles included in this review is shown in Additional File 1.

## Results

The literature search yielded 471 studies, 124 of which were duplicated after the inclusion criteria were checked. The title, abstract, and full text were then examined, and an additional 286 studies were eliminated, as shown in the flowchart (Fig. 1). Finally, 61 studies were included for 4316 *P. falciparum* and 1950 *P. vivax* isolates. Of the 4316 individual isolates infected with *P. falciparum*, 1458 (29.05%) were treated with CQ, 271 (4.3%) with SP, treatment of CQ plus SP with a single dose of primaquine (PQ) 28 (0.73%) for gametocytaemia, 32 (0.83%) received artemether (AM) and PQ, 138 (0.85%) received artesunate (AS) plus SP, 53 (1.37%) received AS–AQ–PQ, 31 (0.8%) received CQ plus PQ, 195 (5.05%) received artemether-lumefantrine (AL), 235 (6.1%) received AS–AQ, 24 (0.62%) received (AS–AQ) plus PQ, 1642 (42.6%) received DHA–PPQ, and 209 (5.41%) received DHA–PPQ plus PQ.

In this review, PQ was administered as a single dose (0.75 mg/kg) on day 3 or PQ single dose of 45 mg on days 0 and 2 [12, 13, 43–46, 64–70]. PQ was administered as a gametocytocide. Of the 1950 individual isolates infected with *P. vivax*, 793 (40.5%) were treated with CQ, 11 (0.65%) were treated with ART-SP, 84 (5%) received CQ plus PYR, 83 (5%) received AQ, 212 (12.6%) received SP, 78 (4.6%) received CQ plus PQ, 19 (1.1%) received HL, 167 (10%) were treated with AS–AQ plus PQ for 14 days (0.25 mg base/kg BW), 164 (9.7%) received DHA–PPQ plus PQ for 14 days (0.25 mg base/kg BW), and 266 (10.9%) were treated with DHA–PPQ [71–84]. In *P. falciparum* observation, patients were followed up for 3 days in one study (n = 119), 28 days in six studies (n = 420), 35 days in one study (n = 114), and 42 days in five studies (n = 993). In terms of *P. vivax* isolates, patients were followed up for 8 days in one study (n = 46), 21 days in two studies (n = 315), 28 days in six studies (n = 407), 42 days in one study (n = 164).

**Fig. 2:**
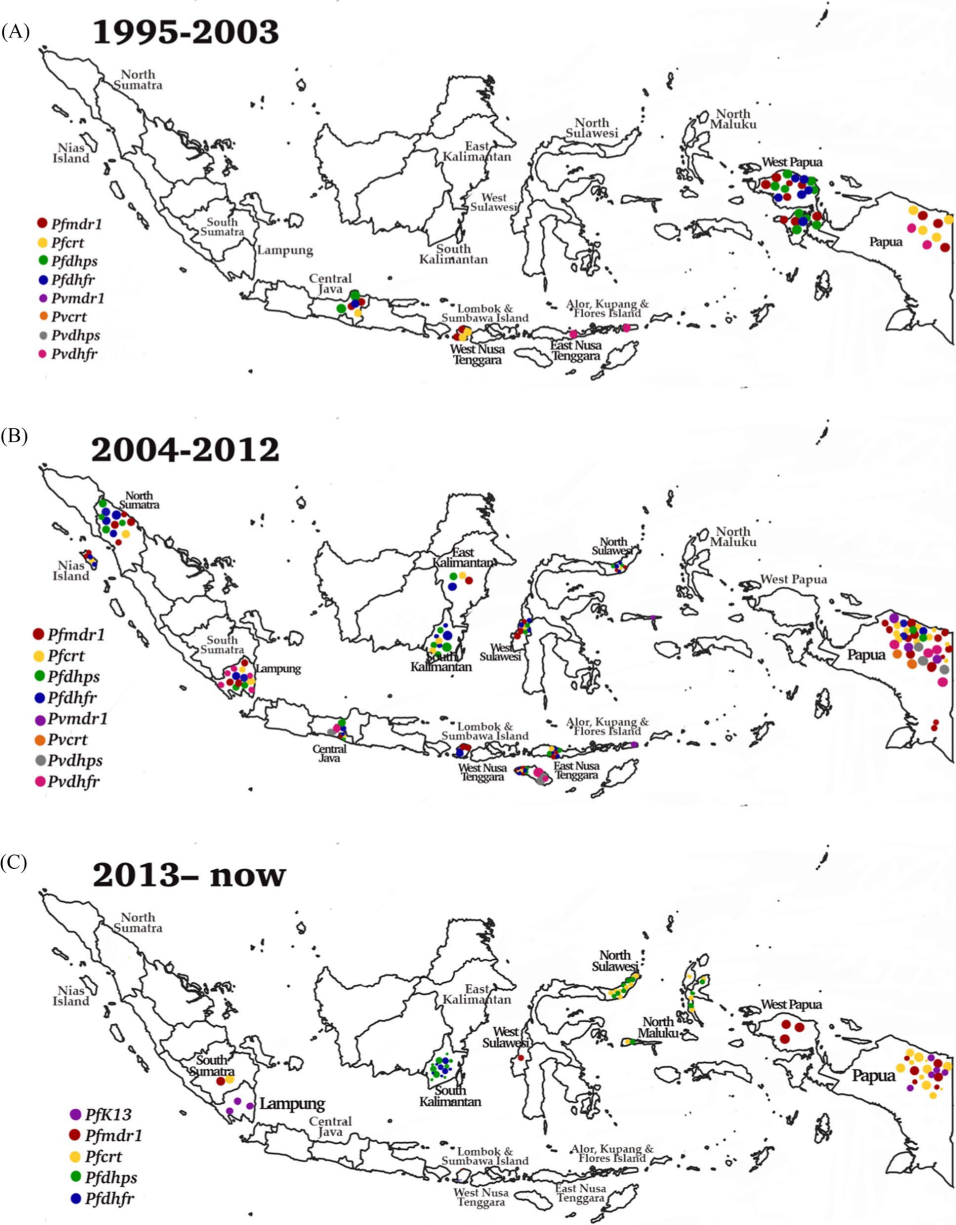
Spatiotemporal map of molecular marker anti-malarial drug resistance (**A**) 1995–2003, (**B**) 2004–2012, (**C**) 2012 to date. Original map was obtained from Natural Earth (https://www.naturalearthdata.com) and modifed according to data from the references

This systematic review included studies conducted in western Indonesia, including North Sumatra, South Sumatra, Nias Island (North Sumatra), Lampung, Central Java, East Kalimantan, South Kalimantan, North Sulawesi, West Sulawesi, Lombok, Sumbawa, and eastern parts, such as East Nusa Tenggara, North Maluku, Alor, Kupang, Flores Island in East Nusa Tenggara, West Papua, and Papua. The dynamics of molecular markers analysed included SNPs in the *pfcrt*, *pfmdr1*, *pfdhfr*, *pfdhps*, and their homologs in *P. vivax*, as shown in Fig. 2. The vast majority of the isolates carried the *pfcrt* (C72S; V73I; M74I; N75D/K; K76T/N; H97L/Y; T152A; S163R; A220S; N326D; T333A/S; I356L/T) and *pvcrt-o* (AAG insertion), *pfmdr1* (N86Y; Y184F; N1042D; S1034C; D1246Y) and *pvmdr1* (Y976F; F1076L), *pfdhfr* (A16V; C59R; S108R; S108N; S108T; I164L) and *pvdhfr* (15S; 49R; N50K; F57L; S58R; T61M; 111L; S117N; S117T; 173F), and *pfdhps* (S436A; A437G; K540E; A581T; A581G; I588G; I588F; A613S/T) and *pvdhps* (383G) in various endemic areas.

From 1995 to 2003, epidemiology studies in molecular markers of anti-malarial drug resistance *P. falciparum* have mostly spread in Papua. Subsequently, from 2004 to 2012, after the ACT recommendation, the molecular marker *P. falciparum* has continuously spread in Sumatra, East Nusa Tenggara, Kalimantan, Sulawesi, and Papua. In terms of co-endemic from 2004 to 2012, molecular markers of anti-malarial drug resistance for *P. vivax* have been detected predominantly compared to other observation periods.

### Distribution of mutant alleles associated with resistance to aminoquinoline

**Fig. 3:**
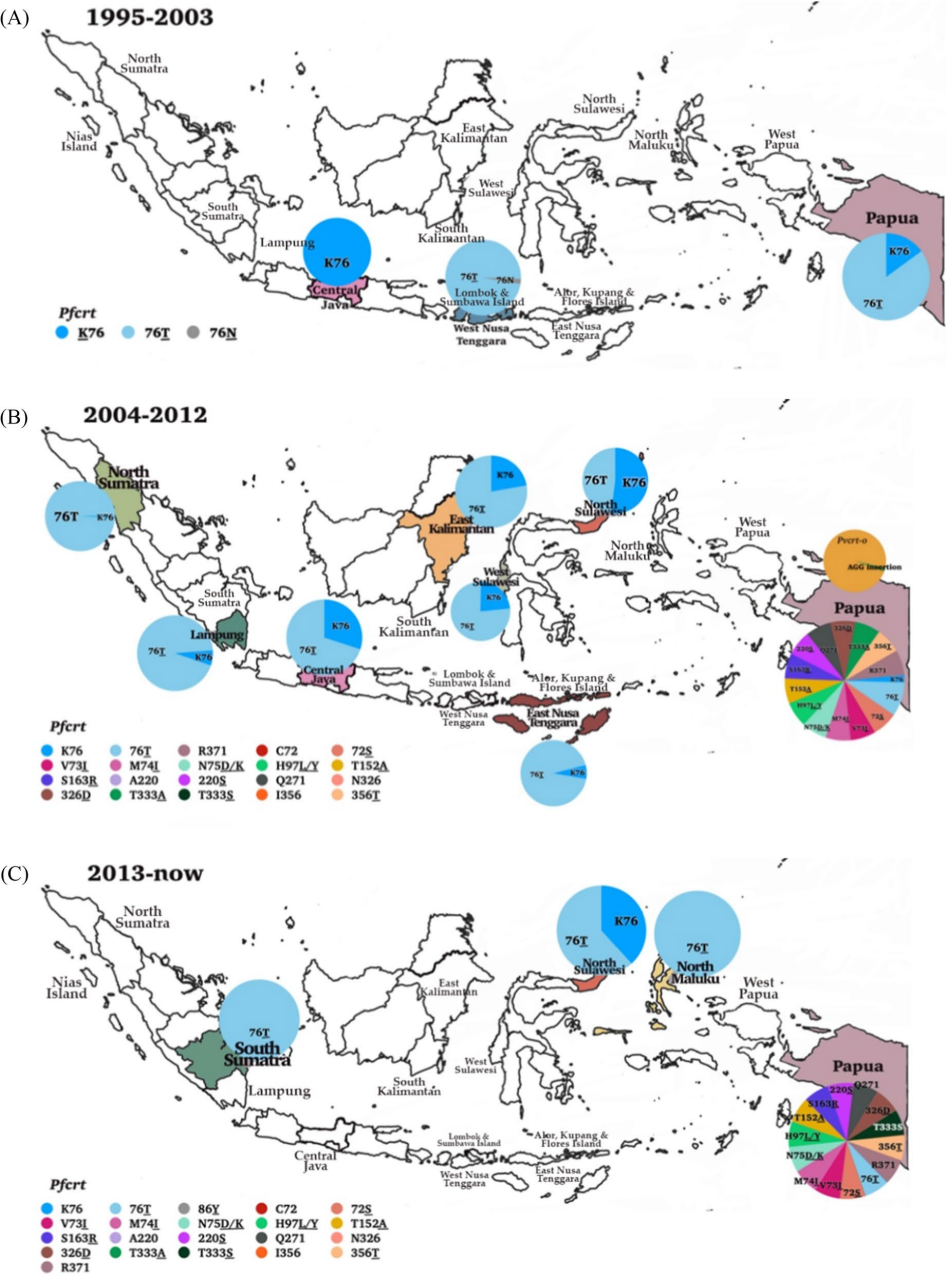
Dynamics map of *pfcrt* and *pvcrt* in three time periods (**A**) 1995–2003, (**B**) 2004–2012 (**C**) 2013 to date. Map source from Natural Earth (https://www.naturalearthdata.com) and modifed according to data from the references

In this study, anti-malarial drug administration to patients included the 4-aminoquinolines, including CQ and AQ, which act as schizontocidal and gametocytocidal. The analysis of 18.3% (789/4316) individual isolates of *pfcrt* from western and eastern parts of Indonesia demonstrated that polymorphism in the *pfcrt* gene, K76**T**, has spread to all sample collection sites in North Sumatra, Nias Island in North Sumatra, South Sumatra, Lampung, North Sulawesi, North Maluku, East Nusa Tenggara, and Flores Island in East Nusa Tenggara during 1995. A highly variant in *pfcrt* SNPs mutation was detected in Papua isolates (Table 2; Fig. 3). Several SNPs formed four *pfcrt* haplotypes, such as C72**S**/V73/M74/N75/K76**T** **haplotype** as the dominant allele being present in 336 of 387 (86.8% of evaluable isolates), C72/V73/M74/N75/K76 being present in 66 of 387 (17.05%), and C72/V73**/**M74**I/**N75**E/**K76**T** being present in 42 of 387 isolates (10.85%). Parasites carrying the C72/V73/M74/N75**/**K76**N** haplotype were the rarest in all study areas, comprising 1 out of 387 in one individual in Lombok (0.25%).

**Table 2:** Dynamics of *P. falciparum* molecular marker genes and putative mutations in Indonesia

Year	Site	Molecular marker	Mutation	Number of mutation (%)	Number of isolate
2002	Lombok [24]	*pfcrt*	K76N	1 (2.1%)	48
			K76T	47 (97.9%)	48
		*pfmdr1*	N86Y	17 (35.4%)	48
2003	Central Java [15]	*pfmdr1*	N86Y	102 (91.9%)	111
			N1042D	5 (4.5%)	111
		*pfdhps*	A437G	39 (35.1%)	111
			K540E	29 (26.1%)	111
		*pfdhfr*	S108N, S108T or C59R, A16V	94 (84.7%)	111
2004	North Sumatra [30]	*pfcrt*	K76T	105 (99.1%)	106
		*pfmdr1*	N86Y	40 (37.7%)	106
			N1042D	2 (1.9%)	106
		*pfdhps*	A437G	1 (0.9%)	106
			K540E	1 (0.9%)	106
		*pfdhfr*	C59R	57 (53.8%)	106
			S108N	87 (82.1%)	106
2005	Lampung [110]	*pfcrt*	K76T	12 (75%)	16
		*pfmdr1*	N86Y	16 (100%)	16
		*pfdhps*	A437G	5 (31.3%)	16
			K540	16 (100%)	16
		*pfdhfr*	C59R	10 (62.5%)	16
			S108N/T	13 (81.3%)	16
	North Sumatra [110]	*pfcrt*	K76T	105 (99.1%)	106
		*pfmdr1*	N86Y	40 (37.7%)	106
			N1042D	2 (1.9%)	106
		*pfdhps*	A437G	1 (0.9%)	106
			K540E	1 (0.9%)	106
		*pfdhfr*	C59R	57 (53.8%)	106
			S108N	87 (82.1%)	106
	Nias, North Sumatra [110]	*pfcrt*	K76T	8 (100%)	8
		*pfmdr1*	N86Y	7 (87.5%)	8
			1042D	0 (0%)	8
		*pfdhps*	K540E	0 (0%)	8
			A437G	0 (0%)	8
		*pfdhfr*	108N/T	5 (62.5%)	8
			16V	0 (0%)	8
	Kutai, East Kalimantan [110]	*pfcrt*	K76T	7 (77.8%)	9
		*pfmdr1*	N86Y	7 (87.5%)	8
		*pfdhps*	A437G	2 (22.2%)	9
		*pfdhfr*	108N/T	4 (44.4%)	9
			C59R	0 (0%)	9
			A16V	0 (0%)	9
	Minahasa, North Sulawesi [110]	pfcrt	K76T	12 (48.0%)	25
		*pfmdr1*	1042D	3 (12.0%)	25
			N86Y	14 (56.0%)	25
		*pfdhps*	A437G	4 (16.0%)	25
			K540E	1 (4.0%)	25
		*pfdhfr*	A16V	0 (0%)	25
			C59R	2 (8.0%)	25
			A16V	0 (0%)	25
			S108N/T	17 (68.0%)	25
	Mamuju, West Sulawesi [110]	*pfcrt*	K76T	10 (76.9%)	13
		*pfmdr1*	N86Y	5 (38.5%)	13
			N1042D`	0 (0%)	13
		*pfdhps*	A437G	1 (7.7%)	13
			K540E	0 (0%)	13
		*pfdhfr*	A16V	2 (15.4%)	13
			C59R	4 (30.8%)	13
			S108N/T	12 (92.3%)	13
	West Sumba District, East Nusa Tenggara [110]	*pfcrt*	K76T	43 (91.5%)	47
		*pfmdr1*	N86Y	19 (40.4%)	47
			1042D	1 (2.1%)	47
		*pfdhps*	A437G	1 (2.1%)	47
			K540E	9 (19.1%)	47
		*pfdhfr*	S108N	27 (57.4%)	47
			C59R	12 (25.5%)	47
	Flores Island, East Nusa Tenggara [110]	*pfcrt*	K76T	13 (100.0%)	13
		*pfmdr1*	N86Y	3 (23.1%)	13
			N1042D	10 (76.9%)	13
		*pfdhps*	A437G	0 (0%)	13
			K540E	0 (0%)	13
		*pfdhfr*	A16V	2 (15.4%)	13
			C59R	3 (23.1%)	13
			S108N/T	11 (84.6%)	13
	Armopa, Papua [110]	*pfcrt*	K76T	8 (61.5%)	13
		*pfmdr1*	N86Y	3 (23.1%)	13
			N1042D	0 (0%)	13
		*pfdhps*	A437G	4 (30.8%)	13
			K540E	0 (0%)	13
		*pfdhfr*	A16V	0 (0%)	13
			C59R	0 (0%)	13
			S108N/T	9 (69.2%)	13
	Kokap, Central Java [110]	*pfcrt*	K76T	14 (70.0%)	20
		*pfmdr1*	N86Y	20 (100%)	20
			N1042D	0 (0%)	20
		*pfdhps*	A437G	1 (5.0%)	20
			K540E	1 (5.0%)	20
		*pfdhfr*	A16V	7 (35.0%)	20
			C59R	7 (35.0%)	20
			S108N/T	18 (90.0%)	20
2006	West Sumba District, East Nusa Tenggara [9]	*pfcrt*	K76T	43 (91.5%)	47
		*pfdhps*	A437G	1 (2.1%)	47
			K540E	9 (19.1%)	47
		*pfdhfr*	S108N	27 (57.4%)	47
			C59R	12 (25.5%)	47
2010	Lampung [163]	*pfcrt*	K76T	46 (100%)	46
	Lombok, Sumbawa, Alor and Kupang islands [113]	*pfmdr1*	N86Y	131 (100%)	131
			S1034C	131 (100%)	131
	West Lombok [113]	*pfdhfr*	S108N	37 (100%)	37
2012	Lampung [164]	*pfmdr1*	N86Y	17 (85.0%)	20
	South Kalimantan [29, 32]	*pfdhps*	A437G	27 (100%)	27
			K540E	9 (33.3%)	27
			A581T	9 (33.3%)	27
			I588G	12 (44.4%)	27
		*pfdhfr*	C59R	24 (88.9%)	27
			S108N/T	27 (100%)	27
			I164L	8 (29.6%)	27
2013	Jayapura, Papua [34]	*pfdhps*	K540E	3 (30.0%)	10
	South Kalimantan [9]	*pfdhps*	A437G	27 (100%)	27
			K540E	9 (33.3%)	27
			A581T	9 (33.3%)	27
			I588F	6 (22.2%)	27
		*pfdhfr*	C59R	24 (88.9%)	27
			S108N/T	27 (100%)	27
			I164L	8 (29.6%)	27
2014	South Sumatra [5]	*pfmdr1*	N86Y	25 (100.0%)	25
		*pfcrt*	K76T	25 (100.0%)	25
	South Kalimantan [9]	*pfdhps*	A437G	27 (100.0%)	27
			K540E	9 (33.3%)	27
			A581T	9 (33.3%)	27
		*pfdhfr*	C59R	24 (88.9%)	27
			S108N/T	27 (100.0%)	27
			I164L	8 (29.6%)	27
2015	North Sulawesi [165]	*pfcrt*	K76T	9 (100.0%)	9
		*pfdhfr*	C59R	3 (33.3%)	9
			S108N/T	6 (66.7%)	9
	Minahasa, North Sulawesi [165]	*pfcrt*	K76T	12 (48.0%)	25
	North Maluku [165]	*pfcrt*	K76T	8 (100.0%)	8
		*pfdhfr*	C59R	1 (12.5%)	8
			S108N/T	6 (75.0%)	8
	Southern Papua [35, 74]	*pfK13*	K13	0 (0%)	65
			Plasmepsin 2–3	0 (0%)	74
	Northwestern Sumatra [150]	*pfK13*	T474A	6 (66.7%)	9
			C580Y	1 (11.1%)	9
2016	Lampung [90]	*pfK13*	G453W	1 (20.0%)	5
			V454C	1 (20.0%)	5
			E455K	1 (20.0%)	5
	Southern Papua [13]	*pfK13*	K13	0 (0%)	65
		Plasmepsin 2	Copy number	0 (0%)	74
	West Papua [106, 167]	*Pfmdr1*	N86Y	35 (81.4%)	43
2017	North Sulawesi [14–16]	*pfmdr1*	N86Y	59 (62.1%)	95

The treatment failure rate for CQ in *P. vivax* infection appeared in 14% of infections among residents of Nias in North Sumatra [75]. Thereafter in 1995, CQ therapeutic failure rate for *P. vivax* and *P. falciparum* was 15% (n = 34) and 30% (n = 37), respectively. Based on Baird et al., among 50% of the individual treated, there was in vivo resistance to CQ, and the weekly 300 mg prophylaxis base tablet of CQ was not effective against *P. vivax* [85]. In West Kalimantan during 1996, Fryauff et al. reported another analysis of the cumulative incidence of therapeutic failure among *P. falciparum* cases, in 1997, representing at day 28 was 7%. In addition, all 20 *P. vivax* parasitaemias were sensitive to chloroquine, and the blood remained clear, except for one case in which an asymptomatic parasitaemia appeared on day 28 [11]. The sample analysis obtained in 1996 by Fryauff et al. revealed parasitaemias cleared initially within four days of beginning supervised chloroquine therapy (25 mg base/kg over a 48-h period), but asexual parasites reappeared within 28 days in 52% (27 of 52) *P. vivax* and 25% (3 of 12) *P. falciparum* cases [43]. In the study between November 1996 and July 1999 in Papua of a 28-day observation, in vivo test revealed clinical resistance to CQ in 79 (74%) of the 107 individuals’ samples [12]. Twenty-eight-day cumulative incidence of confirmed resistance to chloroquine was 56% of infections evaluated. Chloroquine should not be considered adequate for treating acute vivax malaria acquired in this region [86].

The prevalence of the *pfcrt* K76**T**, C72**S**, A220**S**, N326**D**, T333**A**/**S**, and I356**T** reached 93.75% (15 of 16) from 2004 to 2012. Afterward, the mutant prevalence *pfcrt* K76T during the study period 2013–2017 was predominant overall (42 of 42, 100%) in South Sumatra, North Sulawesi, and North Maluku but varied among sites (12 of 25, 48%) to (12 of 13, 92.3%). Other SNPs *pfcrt* mutations such as C72**S**, A220**S**, N326**D**, T333**A**/**S**, and I356**T** were found in 12 of 13, or 92.3% of the cases (Table 2).

**Fig. 4:**
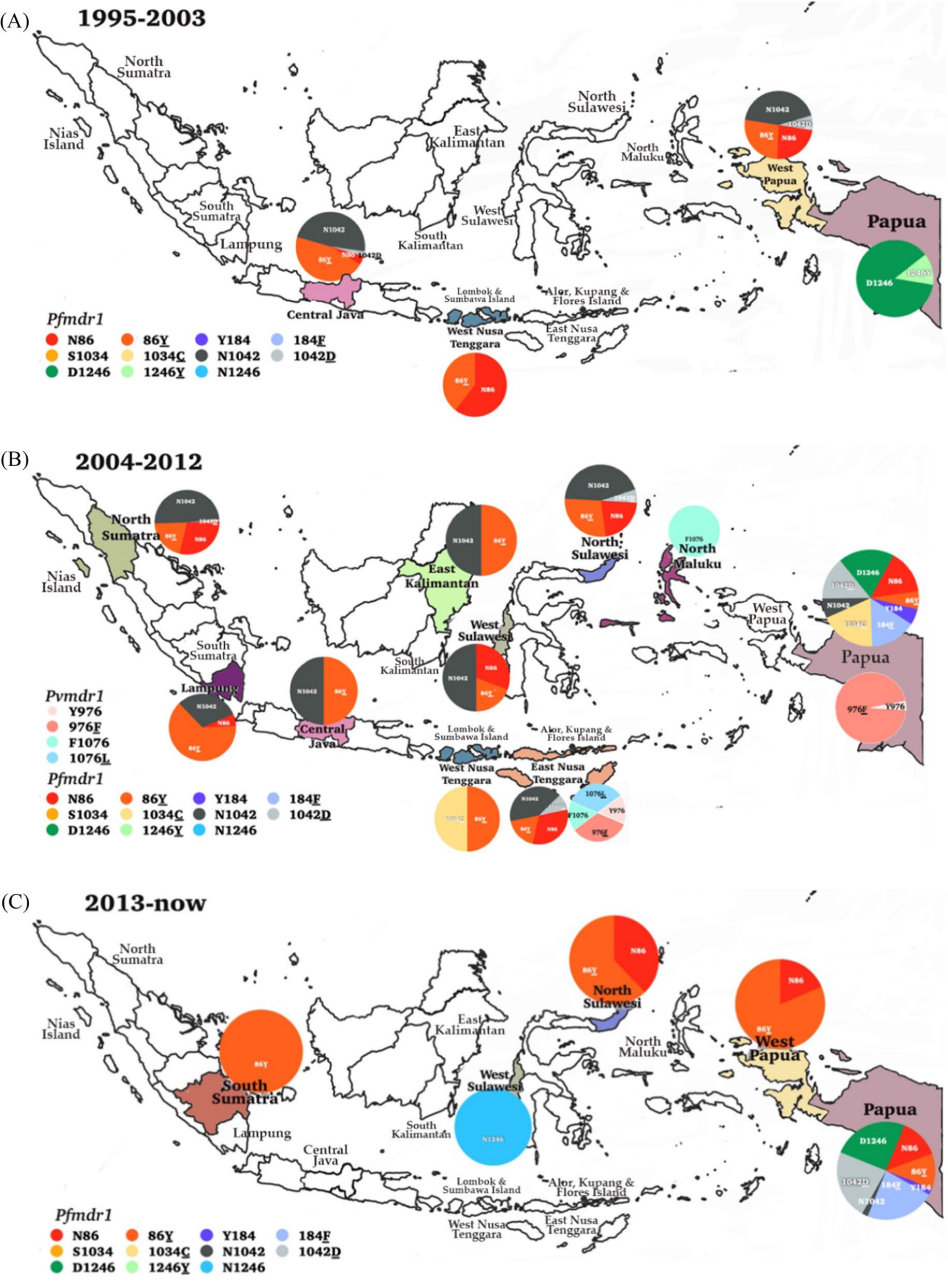
Dynamics map of *pfmdr1* and *pvmdr1* in three time periods (**A**) 1995–2003, (**B**) 2004–2012, (**C**) 2013 to date. Map source from Natural Earth (https://www.naturalearthdata.com) and modifed according to data from the references

Of the 19% of isolates (821/4316) analysed for *pfmdr1*, 35.4% (17/48) isolates carried the *pfmdr1* N86**Y** in 2002, 91.9% (102/111) in 2003, 37.7% (40/106) in 2004, and 62.7% (94/150) in 2005, as shown in Table 2. From 2013 to 2017, this allele was highly prevalent, occurring in more than 80% of isolates in Papua, West Sulawesi, North Sulawesi, and South Sumatra (Fig. 4). The mutant SNP Y184**F** occurred (21 of 35, 60%) in 2004 and, after that, until 2012, varied in Papua (4 of 8, 50%) to (13 of 16, 81.25%). The other *pfmdr1* allele N1042**D** was also present 5/111 (4.5%) in 2003, 2/106 (1.9%) in 2004, and 16/254 (10.3%) in 2005. After 2008, the *pfmdr1* S1034**C** 131/131 (100%) and N86**Y** 131/131 (100%) were found only in Lombok, Sumbawa, Alor, Kupang, and Lampung without any mutation in codon 1042. Polymorphisms at codons D1246**Y** of the *pfmdr*1 gene were observed only in isolates from Papua from 1995 to 2003. Subsequently, from 2004 to 2012, *pfmdr1* SNP mutation changed to wild-type alleles D1246 accompanied by N86**Y**, Y184**F,** and N1042**D** (Fig. 4).

Several authors have reported that N86**Y**/Y184/D1246**Y** and N86/Y184**F**/D1246 haplotypes in *pfmdr1* may be associated with reduced AQ sensitivity and decreased sensitivity to AL, respectively [10, 77]. The N86**Y**/Y184/D1246**Y** haplotype was detected in (24 / 203, 11.8%) before 2008, reflecting the moderate prevalence during AS–AQ deployment in 2004–2008, suggestive reduce AQ susceptibility. Subsequent observation in the following year showed a decreased number of this haplotype (3/203, 1.5%). Thereafter, other haplotypes were detected N86/Y184/D1246**Y** (47 of 203, 23.15%) and N86**Y**/Y184**F**/D1246**Y** (28 of 203, 13.79%).

Regarding *P. vivax*, as shown in Table 3, the highest distribution of SNPs in *pvmdr1* in Indonesia was observed in Maluku Province. The SNP variation in *pvmdr1* before 2008 included F1076**L** 3/4 (75%) and Y976**F** 617/643 (96%). However, since 2008, polymorphism has only been found in codon Y976**F** 123/128 (96.1%) in both areas; Papua and East Nusa Tenggara. Another mutation in *pvcrt*-o with AAG insertion (2.2%) could be found only in Papua persistently before and after DHA–PPQ treatment. Data related to the *pvmdr1* gene copy number were unavailable in any isolates examined (Fig. 4).

**Table 3:** Dynamics of *P. vivax* molecular marker genes and putative mutations in Indonesia

Year	Site	Molecular marker	Mutation	Number of mutation (%)	Number of isolate
2000	Legundi Island, South Lampung [36]	*pvdhfr*	S58R and S117N	5 (41.7%)	12
	Central Java [36]	*pvdhfr*	S58R and S117N	11 (50.0%)	22
			S58R/T61M/S117N	1 (4.5%)	22
			F57L, S58R, T61M, and S117T	2 (12.5%)	16
			15S/F57L/S117T/173F	1 (4.5%)	22
			F57L/S111L/S117T/173F	1 (4.5%)	22
2001	Alor District, Nusa Tenggara [85, 117, 168]	*pvmdr1*	Y976F	2 (66.7%)	3
			F1076L	2 (66.7%)	3
		*pvdhfr*	S58R and S117N	2 (5.6%)	36
2003	Papua [72, 168]	*pvcrt-o*	AAG insertion	1 (2.2%)	46
		*pvmdr1*	Y976F	123 (96.1%)	128
2004	Papua [72, 168]	*pvcrt-o*	AAG insertion	1 (2.2%)	46
		*pvmdr1*	Y976F	123 (96.1%)	128
	Mangole Island, Maluku [77]	*pvmdr1*	F1076L	1 (100%)	1
2005	Papua [72, 168]	*pvcrt-o*	AAG insertion	1 (2.2%)	46
		*pvmdr1*	Y976F	123 (96.1%)	128
2006	Papua [72, 168]	*pvcrt-o*	AAG insertion	1 (2.2%)	46
		*pvmdr1*	Y976F	123 (96.1%)	128
		*pvdhps*	A383G	11 (33%)	34
		*pvdhfr*	S58R/S117N	4 (11.8%)	34
		*pvdhfr*			
	Sumba [169]		S58R/T61M/S117N	1 (2.9%)	34
			F57L/S58R/T61M/S117T	21 (61.8%)	34
			F57L/S58R/T61M/S117T	2 (3.3%)	60
		*pvdhps*	A383G	9%	
	Purworejo [169]	*pvdhfr*	S58R/S117N	6 (60%)	10
			S58R/61M/S117N	0 (0%)	10
			F57L/S58R/T61M/S117T	1 (10%)	10
		*pvdhps*	A383G	15%	
	Lampung [169]	*pvdhfr*	S58R/S117N	26 (96.3%)	27
			S58R/61M/S117N	3 (11.1%)	27
			F57L, S58R, T61M, and S117T	0 (0%)	27
2007	Papua [169]	*pvcrt-o*	AAG insertion	1 (2.2%)	45
		*pvmdr1*	Y976F	123 (96.1%)	128
		*pvdhps*	A383G	33.0%	
		*pvdhfr*	S58R/S117N	4 (11.8%)	34
			58R/61M/S117N	1 (2.9%)	34
			F57L/S58R/T61M/S117T	21 (61.8%)	34
	Sumba [169]	*pvdhfr*	F57L/S58R/T61M/S117T	2 (3.3%)	60
		*pvdhps*	A383G	9%	
	Purworejo [169]	*pvdhfr*	S58R/S117N	6 (60.0%)	10
			S58R/T61M/S117N	0 (0%)	10
			F57L/S58R/T61M/S117T	1 (10.0%)	10
		*pvdhps*	A383G	15%	
	Lampung [169]	*pvdhfr*	S58R/S117N	26 (96.3%)	27
			58R/T61M/S117N	3 (11.1%)	27
			F57L/S58R/T61M/S117T	0 (0%)	27
2008	Papua [169]	*pvcrt-o*	AAG insertion	1 (2.2%)	45
		*pvmdr1*	Y976F	123 (96.1%)	128
		*pvdhps*	A383G	33.0%	
		*pvdhfr*	S58R and S117N	4 (11.8%)	34
			S58R/T61M/S117N	1 (2.9%)	34
			F57L/S58R/T61M/S117T	21 (61.8%)	34
	Sumba [169]	*pvdhfr*	F57L/S58R/T61M/S117T	2 (3.3%)	60
		*pvdhps*	A383G	9%	
	Purworejo [169]	*pvdhfr*	S58R/S117N	6 (60.0%)	10
			S58R/T61M/S117N	0 (0%)	10
			F57L/S58R/T61M/S117T	1 (10.0%)	10
		*pvdhps*	A383G	15%	
	Lampung [169]	*pvdhfr*	S58R/S117N	26 (96.3%)	27
			S58R/T61M/S117N	3 (11.1%)	27
			F57L/S58R/T61M/S117T	0 (0%)	27

### Distribution of mutant alleles associated with resistance to antifolate

Antifolate drug administration to patients in this review study used SP combination therapy. Antifolate drugs are used as anti-malarials through their inhibition of the folate metabolism of the parasite, both in the synthesis and use of folate cofactors. The key enzyme targets are dihydropteroate synthase (DHPS), inhibited by sulfa drugs, and dihydrofolate reductase (DHFR), inhibited by pyrimethamine and cycloguanil.

**Fig. 5:**
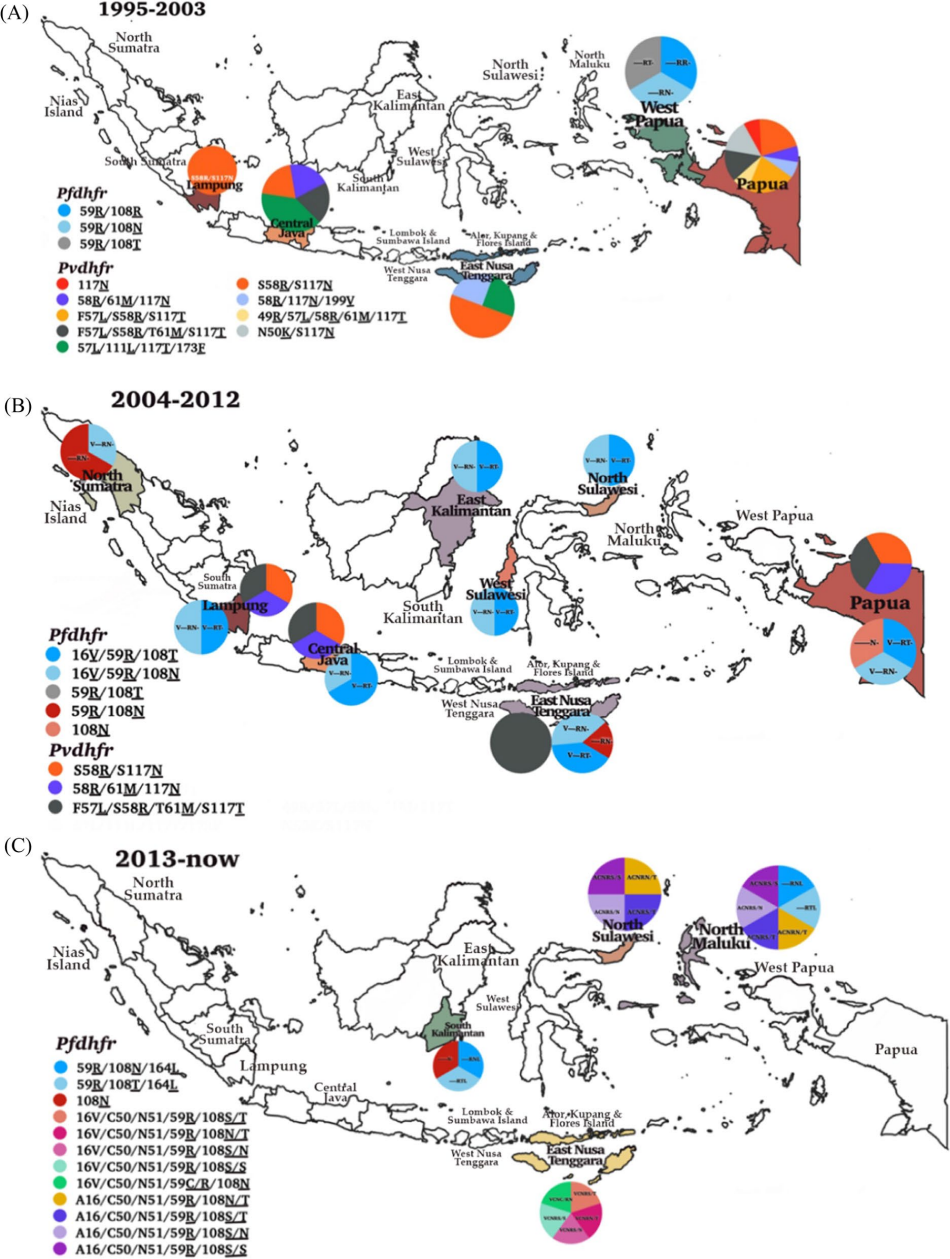
Dynamics map of *pfdhfr* and *pvdhfr* in three time periods (**A**) 1996–2003, (**B**) 2004–2012, (**C**) 2013 to date. Map source from Natural Earth (https://www.natur alearthdata.com) and modifed according to data from the references

Table 2 show that the SNPs related to *pfdhfr* included double mutant C59**R**//S108**N** and A16**V/** S108**T** 94/111 (84.7%) in Central Java during 2003. Other variations including A16**V** 11/46 (22%), N51 17/17 (100%), C59**R** 256/467 (55%), S108**N**/**T** 318/401 (79.3%), and I164**L** 8/27 (30%) were observed in *P. falciparum* isolates before 2008. SNP A16**V** was not observed in North Sulawesi and North Maluku in the following 2008 but increased in the prevalence of S108**N** 167/172 (91.7%), C59**R** 76/98 (62.5%), and I164L 24/81 (29.5%). According to study from Basuki et al. [37], *pfdhfr* haplotypes (based on alleles 16, 51, 59, 108, 164) found in Indonesia were A16/N51/C59/S108**N**/I164 (ANC**N**I), A16/N51/C59/S108/I164 (ANCSI), A16/N51/C59**R**/S108**N**/I164**L** (AN**RNL)**, and A16/N51/C59**R**/S108**N**/I164 (AN**RN**I)**.** In additional observation from 2013 to 2015, four *pfdhfr* haplotypes A16/C50/N51/C59**R**/108 (**N**/**T**), A16/C50/N51/C59**R**/108 (**S**/**T**), A16/C50/N51/C59**R**/108(**S**/**N**), and A16/C50/N51/C59**R**/S108 were found in North Sulawesi and North Maluku (Fig. 5). The double mutant C59**R**/S108**N**/**T** was dominant in North Sumatra, Lampung, Central Java, East Kalimantan, North Sulawesi, West Sulawesi, and Papua. The triple mutant C59**R**/S108**N**/I164**L** was commonly detected in South Kalimantan. In North Sulawesi, there was any change in mutation between 2004 to 2012, and 2013 to date from *pfdhfr* triplet mutation A16**V**/C59**R**/S108**N** and A16**V**/C59**R**/S108**T** confer to *pfdhfr* duplet mutation A16/C50/N51/59**R**/108**N/T**, A16/C50/N51/59**R**/108**S/T**, and A16/C50/N51/59**R**/108**S/N**. In Lampung, Central Java, and East Nusa Tenggara from 1995 to 2003, SNP mutation was detected in *P. vivax* from single mutant, duplet, and triplet. SNP mutation *pvdhfr* quintuple 49R/57L/58R/61M/117T was only found in Papua. Meanwhile, in East Nusa Tenggara, there were no differences in SNPs mutation compared between 2004 to 2012, and 2013 to date (Fig. 5).

**Fig. 6:**
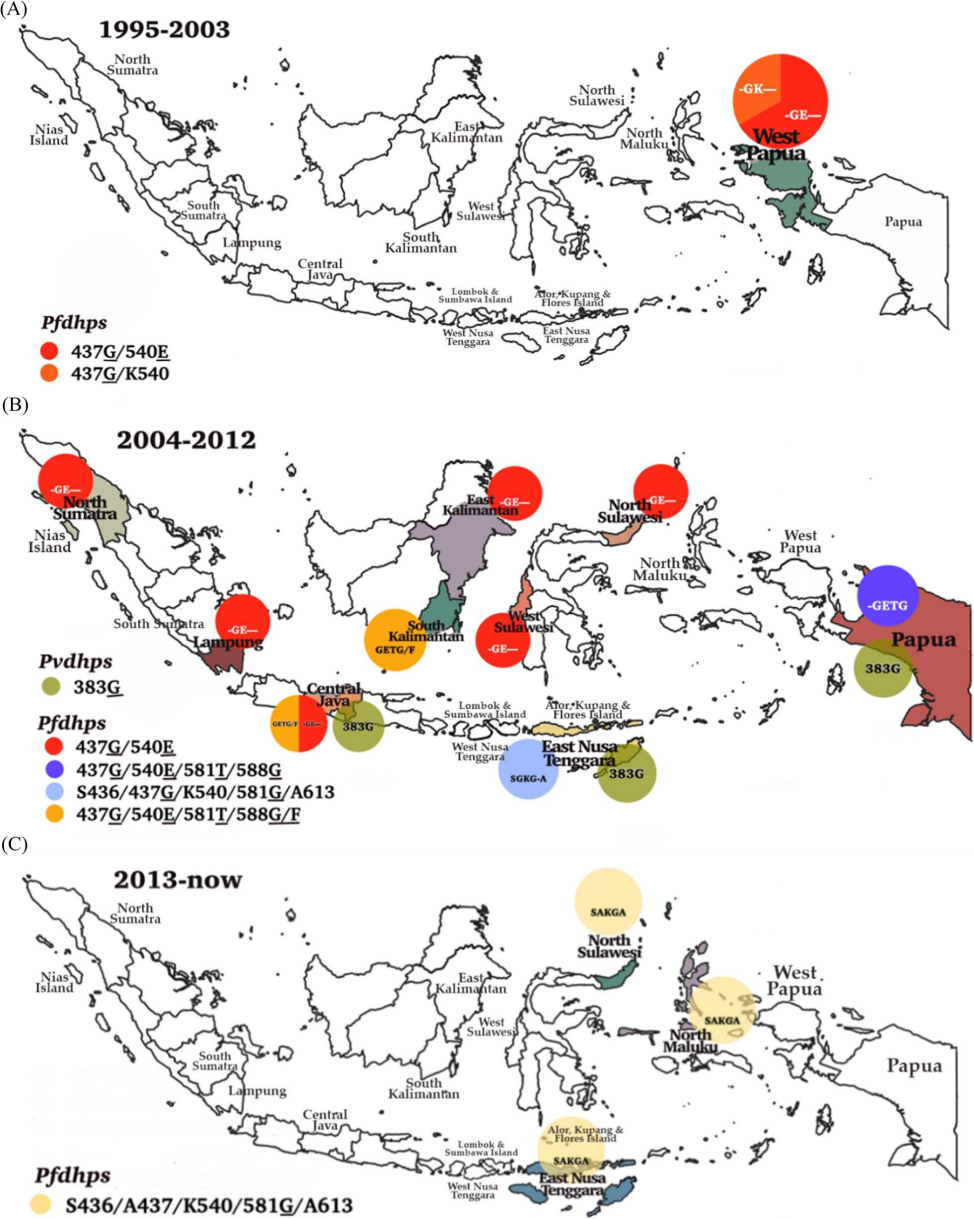
Dynamics map of *pfdhps* and *pvdhps* in three time periods (**A**) 1996–2003, (B) 2004–2012, (**C**) 2013 to date. Map source from Natural Earth (https://www.natur alearthdata.com) and modifed according to data from the references

In addition, SNPs in *pfdhps* included A581**T** 9/27 (33.3%), I588**G** 12/27 (44.4%), and I588**F** 6/27 (22.2%). Before 2008, *P. falciparum* isolates only carried A437**G** and K540**E** (Table 2). The *pfdhps* haplotype S436/A437/K540/A581**G**/A613 was found in North Maluku, North Sulawesi, and East Nusa Tenggara in 2015. Six different mutant alleles S436/A437**G**/K540/A581/A613 (S**G**KAA) (0.03%) in Sumatra, South Kalimantan, Sulawesi, and West Nusa Tenggara; S436/A437**G/**K540/A581**G/**A613 (S**G**K**G**A) (0.03%) in East Java, South Kalimantan, East Kalimantan, and Sulawesi; S436/A437**G**/K540**E**/A581/A613 (S**GE**AA) (0.02%) in East Java, South Kalimantan, East Kalimantan, Central Kalimantan, and West Nusa Tenggara; S436/A437**G/**K540**E/**A581/A613 (S**GE**AA) (588F) (0.04%) in South Kalimantan, Sulawesi, West Nusa Tenggara, and Papua; S436/A437**G**/K540**T/**A581**G/**A613 (S**GTG**A) (0.1%) in East Java, South Kalimantan, East Kalimantan, and Central Kalimantan; and S436/A437**G/**K540**E/**A581**G**/A613 (S**GEG**A) (0.006%) in Jambi, East Java and Central Kalimantan were identified (Table 4) [51]. The *pfdhps* double mutant A437**G**/K540**E** was found predominantly in Papua from 1995 to 2003. The other quadruple mutation accompanied by I588**F** had been identified from 2004 to 2012 isolates from East Kalimantan and Java. From 2013 to date, haplotypes S436/A437/K540/A581**G**/A613 were identified in North Sulawesi, North Maluku, and East Nusa Tenggara. Furthermore, similar variant haplotypes were detected in Sumatra, Java, Kalimantan, West Nusa Tenggara, and Sulawesi (Fig. 6). However, slightly different haplotype variations were observed in the parasite isolates from various research areas in Papua and East Nusa Tenggara. The substitution of I588G**/**F was detected as A437**G**/K540**E**/A581**T** **(GET)** only in South Kalimantan.

**Table 4:** Distribution of *pfdhfr* and *pfdhps* combined haplotypes in Indonesia from 1996 to 2015

No.	Year	Region	pfdhfr	pfdhps	Wild Type	Single mutation	Duplet	Triplet	Quadruplet	Quintuplet	Sextuplet
**1**	1996	West Papua	000RR0	0GE00					59**R**108**R + **437**G**540**E**		
**2**	1997		000RN0	0GE00					59**R**108**N + **437**G**540**E**		
**3**	1998		000RT0	0GE00					59**R**108**T + **437**G**540**E**		
**4**	1999			0GK00							
**5**	2003	Central Java	V00RN0 & V00RT0	0GE00							
**6**	2004	Indragiri Hilir, Riau					AN**RN**I + SAKAA				
**7**	2004	West Nusa Tenggara			SAKAA + ANCSI	ANC**N**I + SAKAA, ANCSI + S**G**KAA	AN**RN**I + SAKAA, ANCSI + S**GE**AA		AN**RN**I + S**GE**AA (588f)	AN**RN**I + S**GTG**A	
**8**	2004	Paser, East Kalimantan					ANCSI + S**G**K**G**A, ANCSI + S**GE**AA			AN**RN**I + S**GEG**A, AN**RN**I + S**GTG**A	AN**RNL** + S**GTG**A
**9**	2004	Pacitan, East Java								AN**RN**I + S**GTG**A, AN**RNL** + S**G**K**G**A, AN**RNL** + S**GE**AA	AN**RNL** + S**GEG**A
**10**	2004	Jayapura, Papua					ANCSI + S**GE**AA				
**11**	2004	North Sumatera	000RN0	0GE00							
**12**	2005			0GE00							
**13**	2005	Indragiri Hilir, Riau					AN**RN**I + SAKAA				
**14**	2005	West Nusa Tenggara			SAKAA + ANCSI	ANC**N**I + SAKAA, ANCSI + S**G**KAA	AN**RN**I + SAKAA, ANCSI + S**GE**AA		AN**RN**I + S**GE**AA (588f)	AN**RN**I + S**GTG**A	
**15**	2005	Lampung	V00RN0 AND V00RT0	0GE00						16**V/**59**R/**108**N + **437**G/**540**E**	
**16**	2005	Nias, North Sumatera	V000RN0 AND V00RT0	0GE00						16**V**/59**R**/108**T + 437G/540E**	
**17**	2005	Kutai, East Kalimantan	V00RN0 AND V00RT0	0GE00							
**18**	2005	Paser, East Kalimantan					ANCSI + S**G**K**G**A, ANCSI + S**GE**AA			AN**RN**I + S**GEG**A, AN**RN**I + S**GTG**A	AN**RNL** + S**GTG**A
**19**	2005	Minahasa, North Sulawesi	V00RN0 AND V00RT0	0GE00							
**20**	2005	Mamuju, West Sulawesi	V00RN0 AND V00RT0	0GE00							
**21**	2005	Flores, East Nusa Tenggara	V00RN0 AND V00RT0								
**22**	2005	Armopa, Papua	V00RN0 AND V00RT0	0GETG							16**V**/59**R**/108**N** + 437**G**/540**E**/581**T** (588**G/F**)
**23**	2005	Jayapura, Papua					ANCSI + S**GE**AA				
**24**	2005	Kokap, Central Java		0GETG							16**V**/59**R**/108**T** + 437**G**/540**E**/581**T** (588**G/F**)
**25**	2005	Pacitan, East Java								AN**RN**I + S**GTG**A, AN**RNL** + S**G**K**G**A, AN**RNL** + S**GE**AA	AN**RNL** + S**GEG**A
**26**	2005	West Sumba District, East Nusa Tenggara	000RN0	SGKG0A					59**R/**108**N + **S436/437**G**/K540/581**G**/A613		
**27**	2006		V00RN0 AND V00RT0	SGKG0A							
**28**	2006	Indragiri Hilir, Riau					AN**RN**I + SAKAA				
**29**	2006	West Nusa Tenggara			SAKAA + ANCSI	ANC**N**I + SAKAA, ANCSI + S**G**KAA	AN**RN**I + SAKAA, ANCSI + S**GE**AA		AN**RN**I + S**GE**AA (588f)	AN**RN**I + S**GTG**A	
**30**	2006	Paser, East Kalimantan					ANCSI + S**G**K**G**A, ANCSI + S**GE**AA			AN**RN**I + S**GEG**A, AN**RN**I + S**GTG**A	AN**RNL** + S**GTG**A
**31**	2006	Pacitan, East Java								AN**RN**I + S**GTG**A, AN**RNL** + S**G**K**G**A, AN**RNL** + S**GE**AA	AN**RNL** + S**GEG**A
**32**	2006	Jayapura, Papua					ANCSI + S**GE**AA				
**33**	2009	Indragiri Hilir, Riau				ANCSI + S**G**KAA					
**34**	2009	Merangin, Jambi									AN**RNL** + S**GEG**A
**35**	2009	West Nusa Tenggara					AN**RN**I + SAKAA, ANCSI + S**GE**AA				
**36**	2009	East Nusa Tenggara					AN**RN**I + SAKAA				
**37**	2009	Banjar, South Kalimantan				ANC**N**I + SAKAA	ANCSI + S**G**K**G**A, ANCSI + S**GE**AA		AN**RN**I + S**GE**AA (588f),, AN**RNL** + S**G**KAA	AN**RN**I + S**GEG**A, AN**RN**I + S**GTG**A	AN**RNL** + S**GTG**A
**38**	2009	Seruyan, Central Kalimantan								AN**RN**I + S**GTG**A, AN**RNL** + S**G**K**G**A, AN**RNL** + S**GE**AA	AN**RNL** + S**GEG**A
**39**	2009	Gorontalo, Sulawesi				ANCSI + S**G**KAA			AN**RN**I + S**GE**AA (588f)		
**40**	2009	Papua, Jayapura			ANCSI + SAKAA		ANCSI + S**GE**AA, AN**RN**I + SAKAA		AN**RN**I + S**GE**AA (588f)		
**41**	2010	Indragiri Hilir, Riau				ANCSI + S**G**KAA					
**42**	2010	Merangin, Jambi									AN**RNL** + S**GEG**A
**43**	2010	West Nusa Tenggara					AN**RN**I + SAKAA, ANCSI + S**GE**AA				
**44**	2010	East Nusa Tenggara					AN**RN**I + SAKAA				
**45**	2010	Banjar, South Kalimantan				ANC**N**I + SAKAA	ANCSI + S**G**K**G**A, ANCSI + S**GE**AA		AN**RN**I + S**GE**AA (588f),, AN**RNL** + S**G**KAA	AN**RN**I + S**GEG**A, AN**RN**I + S**GTG**A	AN**RNL** + S**GTG**A
**46**	2010	Seruyan, Central Kalimantan								AN**RN**I + S**GTG**A, AN**RNL** + S**G**K**G**A, AN**RNL** + S**GE**AA	AN**RNL** + S**GEG**A
**47**	2010	Gorontalo, Sulawesi				ANCSI + S**G**KAA			AN**RN**I + S**GE**AA (588f)		
**48**	2010	Papua, Jayapura			ANCSI + SAKAA		ANCSI + S**GE**AA, AN**RN**I + SAKAA		AN**RN**I + S**GE**AA (588f)		
**49**	2011	Indragiri Hilir, Riau				ANCSI + S**G**KAA					
**50**	2011	Merangin, Jambi									AN**RNL** + S**GEG**A
**51**	2011	West Nusa Tenggara					AN**RN**I + SAKAA, ANCSI + S**GE**AA				
**52**	2011	East Nusa Tenggara					AN**RN**I + SAKAA				
**53**	2011	Banjar, South Kalimantan				ANC**N**I + SAKAA	ANCSI + S**G**K**G**A, ANCSI + S**GE**AA		AN**RN**I + S**GE**AA (588f),, AN**RNL** + S**G**KAA	AN**RN**I + S**GEG**A, AN**RN**I + S**GTG**A	AN**RNL** + S**GTG**A
**54**	2011	Seruyan, Central Kalimantan								AN**RN**I + S**GTG**A, AN**RNL** + S**G**K**G**A, AN**RNL** + S**GE**AA	AN**RNL** + S**GEG**A
**55**	2011	Gorontalo, Sulawesi				ANCSI + S**G**KAA			AN**RN**I + S**GE**AA (588f)		
**56**	2011	Jayapura, Papua			ANCSI + SAKAA		ANCSI + S**GE**AA, AN**RN**I + SAKAA		AN**RN**I + S**GE**AA (588f)		
**57**	2012		N	0	ANCSI + SAKAA		ANCSI + S**GE**AA, AN**RN**I + SAKAA		AN**RN**I + S**GE**AA (588f)		
**58**	2012	Indragiri Hilir, Riau				ANCSI + S**G**KAA					
**59**	2012	Merangin, Jambi									AN**RNL** + S**GEG**A
**60**	2012	West Nusa Tenggara					AN**RN**I + SAKAA, ANCSI + S**GE**AA				
**61**	2012	East Nusa Tenggara					AN**RN**I + SAKAA				
**62**	2012	Banjar, South Kalimantan				ANC**N**I + SAKAA	ANCSI + S**G**K**G**A, ANCSI + S**GE**AA		AN**RN**I + S**GE**AA (588f), AN**RNL** + S**G**KAA	AN**RN**I + S**GEG**A, AN**RN**I + S**GTG**A	AN**RNL** + S**GTG**A
**63**	2012	Seruyan, Central Kalimantan								AN**RN**I + S**GTG**A, AN**RNL** + S**G**K**G**A, AN**RNL** + S**GE**AA	AN**RNL** + S**GEG**A
**64**	2012	Gorontalo, Sulawesi				ANCSI + S**G**KAA			AN**RN**I + S**GE**AA (588f)		
**65**	2012	South Kalimantan	N	GETG/F							
**66**	2013		000RNL								
**67**	2014		000RTL								
**68**	2015	East Nusa Tenggara	VCNRN/T						16**V**/C50/N51/59**R**/108**N/T** + S436/A437**/**K540**/**581**G**/A613		
			VCNRS/T						16**V**/C50/N51/59**R**/108**S/T** + S436/A437/K540/581**G**/A613		
			VCNRS/N						16**V**/C50/N51/59**R**/108**S/N** + S436/A437/K540/581**G**/A613		
			VCNRS/S					16**V**/C50/N51/59**R**/108**S** + S436/A437/K540/581**G**/A613			
			VCNC/RN						16**V**/C50/N51/59C/**R**/108**N** + S436/A437/K540/581**G**/A613		
**69**	2015	North Sulawesi	ACNRN/T					A16/C50/N51/59**R**/108**N/T** + S436/A437**/**K540**/**581**G**/A613			
			ACNRS/T					A16/C50/N51/59**R**/108**S/T** + S436/A437/K540/581**G**/A613			
			ACNRS/N					A16/C50/N51/59**R**/108**S/N** + S436/A437/K540/581**G**/A613			
			ACNRS/S				A16/C50/N51/59**R**/S108 + S436/A437/K540/581**G**/A613				
**70**	2015	North Maluku	ACNRN/T					A16/C50/N51/59**R**/108**N/T** + S436/A437**/**K540**/**581**G**/A613			
			ACNRS/T					A16/C50/N51/59**R**/108**S/T** + S436/A437/K540/581**G**/A613			
			ACNRS/N					A16/C50/N51/59**R**/108**S/N** + S436/A437/K540/581**G**/A613			
			ACNRS/S				A16/C50/N51/59**R**/S108 + S436/A437/K540/581**G**/A613

The novel mutations of *pfdhps* genes K540**T**, I588**G**, and I588**F** were detected as the specifically combined haplotypes (A16/N51/C59**R**/S108**N**/I164 + S436/A437**G**/K540**T**/A581**G**/A613) (AN**RN**I/S**GTG**A), (A16/N51/C59**R**/S108**N**/I164**L** + S436/A437**G**/K540**T**/A581**G**/A613) (AN**RNL**/S**GTG**A), (A16/N51/C59**R**/S108**N**/I164 + S436/A437**G**/K540**E**/A581/A613 (AN**RN**I/S**GE**AA) (588**F**)), and (A437**G**/K540**E**/A581**T** **(GET**) (588**G**)) (Fig. 5).

**Fig. 7:**
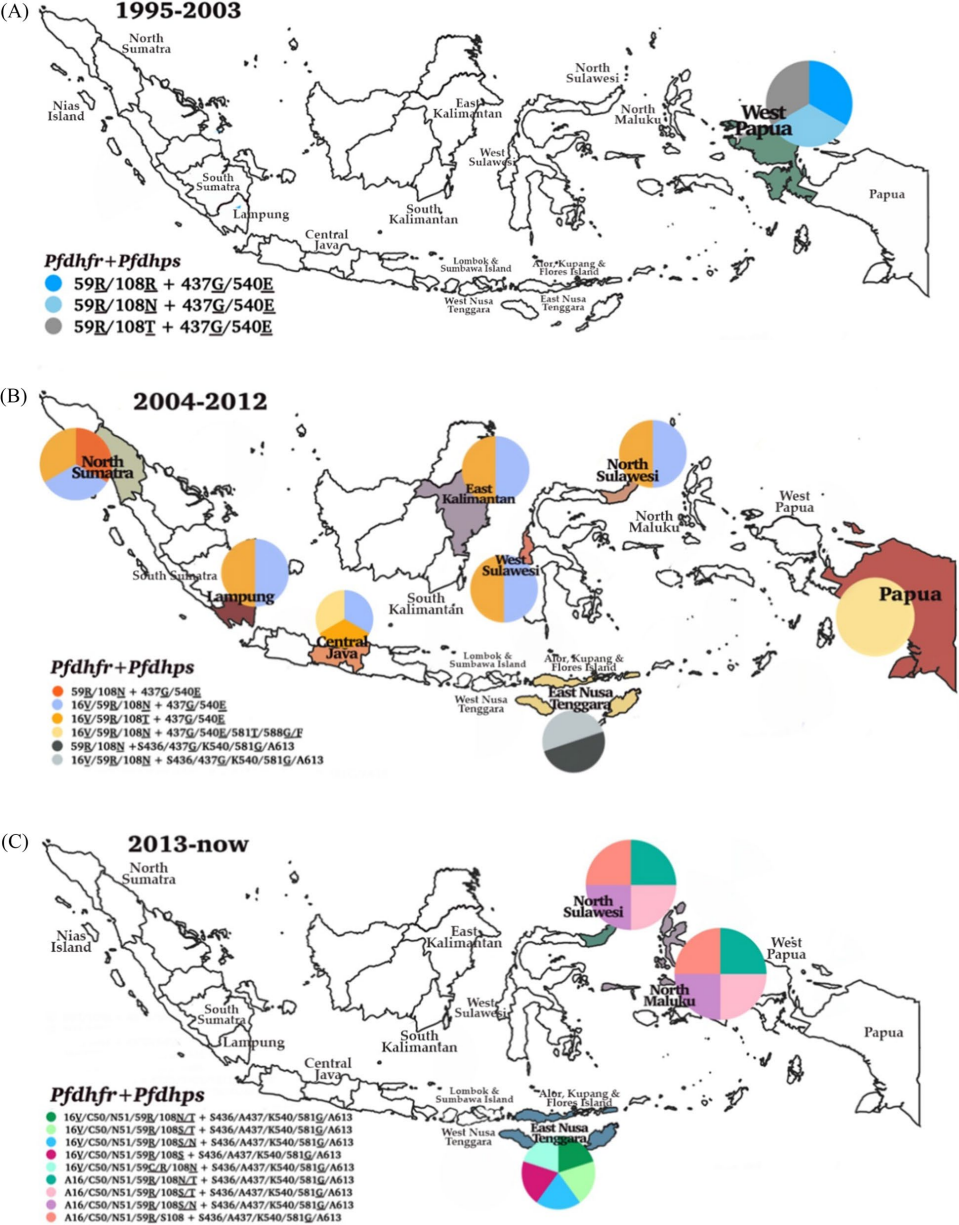
Dynamics map of *pfdhfr/pfdhps* haplotypes in three time periods (**A**) 1996–2003, (**B**) 2004–2012 (**C**) 2013 to date. Map source from Natural Earth (https://www.naturalearthdata.com) and modifed according to data from the references

According to data from Basuki et al. and this review study, the combination of eleven *pfdhr* and eight *pfdhps* haplotypes, a totally of 29 different combined *pfdhfr*/*pfdhps* genotypes were determined; 3 combined haplotypes in 1995–2003, 34 combined haplotypes in 2004–2012 isolates and 10 combined haplotypes in 2013–present isolates. Polymorphisms in the *pfdhfr*/*pfdhps* combined haplotypes were observed to be dominant in West Papua during 1995–2003, Lampung, North Sumatra, Central Java, Middle Kalimantan, South Kalimantan, East Nusa Tenggara, Papua in 2004–2012 and North Sulawesi, North Maluku, East Nusa Tenggara in 2013–present (Table 4). Three quadruple mutants (59**R/**108**R** + 437**G/**540**E**); (59**R/**108**N** + 437**G/**540**E**), and (59**R/**108**T** + 437**G**/540**E**) were observed only in West Papua isolates during 1996–2003, and many different combined haplotypes were found in up to 2015 samples (Fig. 7).

Increased mutations in a combination of haplotypes enhance parasite resistance levels against SP. The parasites with *pfdhfr/pfdhps* quintuple mutant, a genotype marker of SP resistance (A16**V**/C59**R**/S108**N/T** + A437**G**/K540**E**), were found in Lampung, North Sumatra, Central Java, East Kalimantan, West Sulawesi, and North Sulawesi. Previously reported results from Kalimantan (A16/N51/C59**R/**S108**N/**I164 + S436/A437**G/**K540**E/**A581/A613 (588**F**)) (AN**RN**I + S**GE**AA); (A16/N51/C59**R/**S108**N/**I164 + S436/A437**G/**K540**T/**A581**G/**A613) (AN**RN**I + S**GTG**A); (A16/N51/C59**R/**S108**N/**I164**L** + S436/A437**G/**K540**/**A581**G/**A613) (AN**RNL** + S**G**K**G**A); (A16/N51/C59**R/**S108**N/**I164**L** + S436/A437**G/**K540**E/**A581**/**A613) (AN**RNL** + S**GE**AA) and (A16/N51/C59**R/**S108**N/**I164 + S436/A437**G/**K540**E/**A581**G**/A613) AN**RN**I + S**GEG**A were detected from 2004 to 2012 (Fig. 7) [37]. In East Nusa Tenggara, *pfdhfr/pfdhps* quadruple mutants were found persistently during both observation periods, 2004 to 2012 and 2013. The parasites containing quintuple mutants from *pfdhfr*/*pfdhps* have been shown not to respond adequately to SP treatment [87, 88] and were detected mostly in Central Java and Papua from 2004 to 2012. The presence of a single *pfdhfr* mutation (C59**R**) with a single *pfdhps* mutation (K540**E**) accurately predicted the presence of the quintuple mutant [88]. The distribution of *pfdhfr* and *pfdhps* combined haplotypes in Indonesia is presented in Table 4.

Until 2008, SNPs of the *pvdhfr* in *P. vivax* included the double mutant (duplet) S58**R**/S117**N** 90/182 (49.5%), triplet (S58**R**/T61**M**/S117**N**) 6/137 (4.4%), (F57**L**/S58**R**/ S117**T**) 1/22 (4.5%), and quadruplet (15**S**/F57**L**/S117**T**/I173**F**) 1/22 (4.5%), (F57**L**/S111**L**/S117**T**/I173**F**) 1/22 (4.5%) (Table 3) [17, 18]. Quadruple mutations (F57**L**/ S58**R**/ T61**M**/S117**T**) were predominant in Central Java, Papua, Sumba, Purworejo, and Lampung. [89] Finally, *pvdhps* has a less frequent variation of haplotypes with codon involvement in position at 383G before and after 2008, with a prevalence of 19%.

### Distribution of mutant alleles associated with resistance to artemisinin and its drug partner in Indonesia

Initially, AS–AQ was used in Indonesia from 2004 to 2008 as ACT's first-line treatment, until many resistance to this ACT was reported [48–50]. Since 2008, the ACT regimen has changed to DHA–PPQ. The ACT consists of a potent ART component classified as sesquiterpene lactone [51]. In any 1469 *pfK13* isolates examined, no mutation associated with ART resistance was found. Other SNPs mutation *pfK13* genes such as G453**W** (20%), V454**C** (20%), E455**K** (20%), and T474**A** (2.6%) were detected during 2015–2016. By concerning the ART partner drug, an increase in the copy number of the *pfpm2* (3 of 6, 50%) gene was found among 808 isolates that survived the DHA–PPQ treatment in Papua [71, 90], but was not associated with PPQ resistance. This result demonstrates that DHA used in ACT is still highly productive in suppressing parasite density. Another study described a slight decline in PPQ susceptibility, although it did not appear to have reached clinically significant levels [91]. During observation periods, no 8 SNPs mutation in *pvK12* associated with ART resistance, such as M448, T517, F519, I568, S578, D605, and D691, and L708, has been found in Papua and Jambi. *P. vivax* TES in Keerom and Merangin, Jambi province, revealed 100% ACPR of total analysed cases [61].

## Discussion

This review analysed the genotypic patterns of *P. falciparum* and *P. vivax* isolates across wide geographic regions in Indonesia since 1991, when the MoH changed the first-line anti-malarial drugs from CQ to SP, then continued in 2004 with the use of ACT. It is the first longitudinal genotypic profile documenting the molecular marker of anti-malarial drug resistance of co-endemic *P. falciparum* and *P. vivax* analysis over 30 years in Indonesia [5, 92]. Therefore, this study review is expected to give comprehensive information through parasite genetic diversity patterns in the context of epidemiologic investigations.

The K76**T** of the *pfcrt*, a determining SNP that distinguishes resistance to CQ, was found in most *P. falciparum* isolates collected between 1991 and 2004 [12, 93, 94]. Mutations in the *pfmdr1* gene can occur at several codon positions such as 86 (asparagine to tyrosine), 184 (tyrosine to phenylalanine), 1034 (serine to cysteine), 1042 (asparagine to aspartic acid), and 1246 (aspartic acid to tyrosine) [95]. SNP mutation at codon D1246**Y** was only observed in Papua. Studies on the *pfmdr1* gene have identified N86**Y** and Y184**F** being more frequent in Asian and African parasites, whereas S1034**C**, N1042**D**, and D1246**Y** are more common in South American parasites [88]^97^. However, another study could find it in Asia [98]. This spread of South American alleles could explain the possibility of importing parasite isolate from its location, and a hard selective sweep may induce a higher level of mutant allele prevalence [99]. The isolates carried SNPs in *pfmdr1* associated with CQ. resistance, such as N86**Y** 644/968 (67%), N1042**D** 191/558 (34.2%), and S1034**C** 131/334 (39.2%) [100]. The 184F allele is slowly disappearing in Southeast Asia, including Sumatra, Indonesia [101], except in western Cambodia and eastern Thailand [101]. Overall, the genotypic profiles of the *P. falciparum* isolate reflect a continual selective pressure by CQ and other similar drugs, such as QN, AQ, and PPQ, on the isolates in Indonesia. However, CQ is no longer used for *P. falciparum* treatment. That low prevalence of *pfmdr1*’s 184F mutation was due to QN, AQ, and PPQ exposures [102–104]. In addition, it is significant to recognize the interesting evidence that ART selects for the *pfmdr1* N86/Y184**F** haplotype in vivo [94] and in vitro [104] experiments. The selective impact of ACT supports the *pfmdr1* haplotype N86/Y184**F**, originally explained in an African study [105] and Papua [104, 106]. The *pfmdr*1 N86**Y** allele was once widespread in Southeast Asia, but it has slightly declined in frequency as CQ and AQ have been withdrawn [54]. The change to N86 resulted in a three- to four-fold increase in the IC_50_ values for LUM, MQ, and DHA. Codons N86**Y**, Y184**F,** and D1246**Y** are uniquely associated with sensitivity to LUM and AQ in sub-Saharan Africa [107]. Another study in Tanzania observed a high prevalence of *pfmdr1* N86**Y**, Y184, and 1246**Y** in patients who failed treatment with AQ. Longitudinal cohort studies in Africa showed that the SNPs mutation *pfmdr1* at codons N86**Y**, Y184**F**, and D1246**Y** is associated with AL or AS–AQ drug pressure [108, 109]. Meanwhile, Uganda detected a high prevalence of *pfmdr1* N86, Y184**F,** and D1246 alleles after treatment with AL [89]. A dramatic fall in the prevalence of N86**Y** was also detected in Nias Island in North Sumatra, from 100% in 2003 [110] to 31.4% in 2005 [105, 111]. However, it is also related to high *pfcrt* K76**T** mutant prevalence [11]. All *P. falciparum* isolates from Central Java possessed a mutant allele K76**T** of the *pfcrt* gene paired with the N86**Y** or D1042 allele of the *pfmdr1* gene [15]. SNP mutation of *pfmdr1* D1246**Y** allele might reduce the chloroquine 50% inhibitory concentration (IC_50_) [99]. It is in line with the surveys conducted in the same area, which observed resistance of *P. falciparum* to CQ, QN, and MQ by either in vivo or in vitro drug resistance tests [75].

CQ resistance in *P. vivax* has spread in all the countries since 1989 [88]. According to previous *in-vivo* and drug analysis studies in Papua, Sumatra and Sulawesi observed high grade and frequent CQ-resistant in *P. vivax* isolates [75, 112–115]. A study in northeastern Papua [116] and Tjitra et al.[116] in eastern Indonesia showed that the CQ failure rate reached more than 50%. In contrast, a later study by Asih et al. in Sentani Papua in 2007 [114] observed an estimation of a failure rate of 17%. This significant difference might be because naturally acquired immunity by persistent infection among indigenous residents in Sentani contributed to a sharp decrease in failure rate. Another mechanism, such as bottle neck, was greater in *P. falciparum* than in *P. vivax*. It was shown by the adaptation of one minor subpopulation (K2) among 4 subpopulations accounting for 100% of infection in late 2016–2017 [92]. This bottleneck led to the decreased allelic richness, the near fixation of a few alleles, and missing pre-existing alleles [63].

Observations on *P. vivax* isolates identified several SNPs in *pvmdr1*, such as F1076**L** 3/4 (75%) and Y976**F** 617/643 (96%). Brega et al.[117] identified the *P. vivax* orthologue of the *pfmdr1* gene (*pvmdr1*), which was shown to have a role in the drug resistance of *P. falciparum*. The Y976**F** alteration was responsible for a 1.7-fold higher IC_50_ to CQ in Thai isolates [72]. In a study between 2003 and 2006 by Suwanarusk et al.[72], Y976**F** mutation was significantly prevalent in Indonesian isolates that almost reached fixation (96%, 24/25). Two polymorphisms, *pvmdr1* Y976**F** mutation and insertion in the 1st exon (amino acid substitutions 10) of *pvcrt-o* were associated with in vitro CQ susceptibility and a significant increase in CQ IC50. Another study reported the identification of the *pvmdr1* 976 and 1076 mutation in a small number of Thai and Indonesian isolates without in vitro and clinical correlates [117]. The *pvmdr1* Y976**F** mutation, combined with quadruple mutant, refers to *pvdhfr* sequences such as F57**L**/S58**R**/T61**M**/S117**T**, F57**L**/S111**L**/S117**T**/173**F**, and 15**S**/F57**L**/S117**T**/173**F** correlated with treatment failure following AQ plus SP [118].

In this review, *pfdhfr* and *pfdhps* SNPs mutation included several mutant alleles, such as A16**V**, N51**I**, C59**R**, S108**N**/**T**, I164**L**, and S436**F**, A437**G**, K540**E**, A581**T**, A581**G**, and I588**F**/**G**, respectively. The proportion of *pfdhfr* codon position at 108 in various locations in Indonesia has been determined, including double mutant C59**R**/S108**N** and A16**V**/S108**T** (84.7%) in Purworejo-Central Java [14], single mutant S108**N** (71.2%) in Alor-East Nusa Tenggara [114], and double mutant S108**N**/S108**T** (81.3%) in Lampung [74]. The emergence of *pfdhfr* SNP mutation A16**V** is also particularly interesting in resistance to cycloguanil, although this drug has never been used in Indonesia. The highest prevalence of this SNP mutation in Central Java might be due to the involvement of other drugs with similar action to cycloguanil, such as trimethoprim, which is commonly used in combination with sulfamethoxazole for the treatment of bacterial diseases. Another explanation is the presence of the parasite isolates imported from an area where the drug has been applied [119]. However, *pfdhfr* SNP I164**L** mutation was discovered only in South Kalimantan between 2012 and 2014, and *pfdhfr* N51 was detected in North Sulawesi and North Maluku. The *pfdhfr* mutant allele at codons 50 and 51 was absent in the samples examined in Indonesia. Exclusively, two new *pfdhfr* SNP mutations in low-parasitemia Bolivian isolates were detected as a point mutation at codons 50 and 164, showing the continuous establishment of these polymorphisms in a restricted area [120]. *Pfdhfr* mutant allele C50**R** is detected in association with N51**I** and S108**N** in South America and confers midlevel resistance. The *pfdhfr* SNPs mutation S108**N**, N51**I**, and *pfdhps* SNP mutation A437**G** were as the “primary anti-folate resistance mutations,” meanwhile the *pfdhfr* SNPs mutation C50**R**, I164**L,** and *pfdhps* SNP mutation K540**E**, and A581**G** were as the “secondary anti-folate resistance mutations [121].

In Madagascar and Thailand, two haplotypes have been described as *pfdhfr* triple mutants N51**I**/S108**N**/I164**L** and C59**R**/S108**N**/I164**L** might induce six–ten fold higher IC_50_ to PYR [122, 123]. The *pfdhfr* double mutants N51**I**/S108**N** or C59**R**/S108**N** induced PYR resistance 2–16-fold higher than *pfdhfr* single mutant S108**N** [124]. In contrast with another study, the *pfdhfr* single mutant S108**N** conferred a 100-fold increase in resistance to PYR [120, 125]. The *pfdhfr* triple mutants A16/N51/C59**R**/S108**N**/I164**L** and *pfdhps* double mutants S436/A437**G**/K540**E/**A581/A613 were observed in all study sites except in North Maluku and North Sulawesi.

Mutations in S436**A**/**F**, A437**G**, K540**E**/**T**, A581**G**, I588**F**, and A613**S**/**T** in the *pfdhps* gene have been linked to SX resistance [37, 69]. In *P. falciparum*, the resistance rate will increase when the A437**G** mutation is combined with the different mutant allele K540**E** [29]. Another study also reported that a combination between I588**F** and K540**E** mutations could increase the SX resistance [55]. Isolates *P. falciparum* from Purworejo, Central Java, carried multiple mutations in the *pfdhps* A437**G** (35.3%) and K540**E** (26.5%) genes, which might suggest the wider use of the second-line SP anti-malarial following withdrawal of CQ [14]. Another *pfdhps* study [126] reported that point mutations at A437**G** and K540**E** are responsible for SX resistance. It was considered that the point mutation at 437 is the first event and reduced response to SX. A molecular study in Malaysia [127] reported that 87% of isolates had a triple mutation in *pfdhfr,* and all isolates had point mutation at *pfdhps codon* A437**G**, accompanied by 81% point mutation at codon A581**G,** indicating decreased responsiveness of SX. Mutations at A581**G** and A613**S** in the background of A437**G** were associated with high clinical resistance to SP in Thailand and India [128]. A previous African study demonstrated that resistance to SP in vivo was related to three mutant alleles, such as S108**N**, N51**I**, and C59**R,** in the *dhfr* gene with or without mutant alleles A437**G** and K540**E** *dhps* gene [129].

The *P. vivax* isolates carried double mutant (S58**R** + S117**N**) 11/22 (50%) and quadruplet mutant (F57**L**, S58**R**, T61**M**, and S117**T**) 2/16 (12.5%), (15**S**/F57**L**/S117**T**/I173**F**) 1/22 (4.5%), (F57**L**/S111**L**/S117**T**/I173**F**) 1/22 (4.5%) [14] have been linked to resistance to PYR [107]. In *P. vivax*, nonsynonymous SNPs that alter amino acid positions 49, 57, 58, 61, 117, and 173, corresponding to similarly homologous positions in *P. falciparum*, have been shown to confer resistance to PYR [130]. The analogous SNP mutation *pfdhfr* S108**N** with the *pvdhfr* SNP mutation S117**N** conferred approximately 4000- and approximately 1600-fold increased resistance to PYR and cycloguanil, respectively, compared to the wild-type *pvdhfr* [131]. *Pvdhfr* SNP mutation S117**N** could increase the IC_50_ of PYR by more than 80 times [85]. Additionally, the single mutated allele S117**N** was detected at a high frequency in Turkey and Azerbaijan sample isolates, areas where antifolate drug pressure or resistance is not obvious as the first-line treatment in these areas. It occurs because the areas are still use a combination of CQ-PQ [132]. According to this important role of S117**N**, it is assumed that the S117**N** mutation is the first step in the drug resistance selection process [132] and has been strongly associated with SP resistance in areas with extensive use of SP [31, 130]. The double mutant *pvdhfr* S58**R**/S117**N** was 10- to 25-fold less resistant than the S117**N** [131]. In the *pvdhfr* gene, 20 non-synonymous mutations have already been identified [133, 134]. It is contrary to the availability of data from Indonesia, which is still limited. Previous data from Lampung showed a triple mutation in this area [135], and a quadruple mutant F57**L**/S58**R**/T61**M**/S117**T** was found in Papua. This quadruple mutation confers higher resistance to SP than the mutant allele encoding a double mutation (S58**R**/S117**N** or N50**K**/S117**N**) [78]. In 13 of the 16 isolates from Southeast Asia, residues 58 and 117 are implicated in PYR resistance [136, 137]. Triple mutations were found exclusively in Thai parasites. In this study, parasites harbouring triple mutations at F57**L**/S58**R**/ S117**N** are associated with high levels of SP resistance and cleared significantly more slowly in *P. vivax* than those with double mutations of S57**R**/S117**N** [138].

This review found no parasite clearance delay or mutation in the *pfk13* gene associated with ART resistance in any isolates examined after ACT treatment [111, 119, 139, 140]. The *pfK13* propeller domain polymorphisms have been associated with decreased sensitivity to ART in Southeast Asia and have arisen separately in Cambodia and Myanmar [59, 141–143]. The eight non-synonymous mutations observed in Southeast Asia and China, including the F446**I**, N458**Y**, N537**D**, R539**T**, I543**T**, P553**L**, P574**L**, and C580**Y** are related to *P. falciparum* resistance to AS monotherapy or ACT on day 3 [144]. Analyses of the *P. falciparum* genotypes in eastern Indonesia identified another SNPs in the *pfK13* gene, G497**V**, in 0.9% of the 106 samples from Sumba [144]. In western Indonesia, the other SNPs of the *pfK13*, including G453**W**, V454**C**, and E455**K**, were detected in 20% of the isolates. Previous molecular investigations of clinical isolates of *P. falciparum* collected from DHA–PPQ clinical efficacy trials during 2015 and 2016 revealed no *kelch13* polymorphism associated with ART resistance [90]. Nevertheless, late treatment failures related to resistance to partner PPQ were increasingly detected in Papua. Analyses of the copy number of the *pfplasmepsin* 2–3 gene revealed several recurrent isolates that carried an increased copy number of the *pfplasmepsin* 2–3 gene but still failed to identify any association with PPQ resistance. The results suggested that PPQ resistance had slowly emerged among the field isolates and might indicate the preparation of alternative partner drugs to replace PPQ [55]. In another study, *pfpm*2 CNVs did not result in PPQ resistance in vitro [145]. In Cambodia, the *pfplasmepsin* gene cluster showed 2–3 amplification as an important molecular determinant of PPQ resistance in *P. falciparum* [54].

Until now, no cases of *P. vivax* resistance to ACT have been reported in South Pacific and Southeast Asia. However, several studies have monitored the *P. vivax* ortholog of *pfk13*, *pvk12* for polymorphisms that might lead to ART resistance [144]. While several polymorphisms have been found in *pvkelch12*, with low frequencies and limited polymorphisms, such as V552**I**, K151**Q**, and M124**I** (7 of 734, 1%) [146–148] Study in Indonesia also found no polymorphism associated with ART resistance [61]. These results suggested a lack of strong selection pressure from ART on *pvk12* [144, 147]. Drug pressure with ART in the GMS was not related to signatures of selection for mutations in the *Pvk12,* and additional observations, including analysis of associated clinical data from these regions, could further clarify current findings [148].

The orthologous gene for *Pfpm 2/3* in *P. vivax* is *Pvpm4,* identified and mapped for *P. vivax* located on chromosome 13 with a sequence length of 1353 bp [11]. Recently, genetic variation in *pvpm4* I165V has been reported in Malaysia, Thailand, and Indonesia. Unfortunately, this mutation is unlikely to be associated with PPQ drug resistance since its frequency was not associated with the level of PPQ drug pressure. Meanwhile, *pvpm4* amplification was not observed in 141 *P. vivax* field isolates from Thailand and Cambodia [148]. Nowadays, the genetic diversity of *P. vivax* cases from Timika, Papua Indonesia, was detected to be higher than in *P. falciparum*. This result demonstrated the greater refractoriness of *P*. *vivax* to control measures and the risk of distinct parasite subpopulations persisting in the community undetected by passive surveillance [12]

ART resistance is considered to have emerged due to high rates of private-sector self-medication, presumptive fever treatment, misdiagnosis, and the unregulated use of anti-malarial agents, including low-quality ACT medicines [142, 149]. Genetic markers of *pfk13*, *pfcrt,* and *pfmdr1* were diverse, corresponding to variations in populations' transmission levels, treatment-seeking patterns, access to medical care, and use of antimalarials [150]. In Indonesia, the procurement and deployment of DHA–PPQ are strictly regulated by the MoH. The drugs are provided only in carefully selected government-run health institutions and private facilities capable of confirming the malaria diagnosis through microscopy or rapid diagnostic tests. As a result, DHA–PPQ is still highly productive in treating uncomplicated malaria cases. However, in some areas, the second line of ACT should anticipate the increasing cases of DHA–PPQ late treatment failure [74].

Resistance can be caused by using drugs that do not meet the norms, which will encourage the emergence of *Plasmodium*, which is treatment-resistant. If it happens, the trend of increasing parasitic drug resistance in malaria-endemic areas will increase, which is a cause of malaria’s high morbidity and mortality. It indicates that there are still obstacles to the implementation of the provision. Therefore, serious consideration should be given to identifying the demographics of resistance to anti-malarial drugs to help modify chemotherapeutic treatment plans that effectively prevent further development of the resistance and mitigate or eliminate malaria transmission in the districts. The unique characteristics of SNPs in each *pfmdr1*, *pfdhfr*, *pfdhps*, and their orthologue in *P. vivax* play a major role in driving anti-malaria treatment failure. It can help guide the country’s anti-malarial policy for using ACT. The presence of several changes in *pfk13* in the parasite population is of concern and highlights the importance of further evaluation of parasitic ART susceptibility in Indonesia. Although additional efficacy studies are needed, DHA–PPQ appears to be an effective treatment for *P. falciparum* and *P. vivax* infection.

## Conclusion

Summarily, polymorphism genes related to resistance to CQ, SP, and recently ACT, were examined in *P. falciparum* and *P. vivax* field isolates from Indonesia. The findings implied that the prevalence of altered genotypes remained dominant more than 20 years after CQ was removed from this region. The frequency distribution of molecular markers among the *P. falciparum* and *P. vivax* isolates indicated that the currently recommended anti-malarial drug DHA–PPQ is still effective in treating uncomplicated falciparum and vivax malaria. The unique characteristics of SNP haplotypes in each *pfcrt*, *pfmdr1*, *pfdhfr*, *pfdhps*, and its orthologue in *P. vivax* played a key role in driving anti-malarial treatment failure.

## Supplementary Information

**Additional file 1.**

## Data Availability

The datasets used and/or analysed during the current study are available from the corresponding author on reasonable request.
