# Supplementary material for: Drug resistance of Plasmodium falciparum and Plasmodium vivax isolates in Indonesia
Source: Malar J. 2022 Nov 28;21:354. doi: 10.1186/s12936-022-04385-2 (PMC9703442; doi:10.1186/s12936-022-04385-2)
Supplement: Supplementary file 1 — Additional file 1. [file 12936_2022_4385_MOESM1_ESM.docx]

**SUMMARY OF 61 REFERENCES LIST**

| **No.** | **Ref. No.** | **Author** | **Title** |
| --- | --- | --- | --- |
| **1** | **52** | Price RN, Hasugian AR, Ratcliff A, et al. | Clinical and pharmacological determinants of the therapeutic response to dihydroartemisinin-piperaquine for drug-resistant malaria. Antimicrob Agents Chemother. 2007;51(11):4090-4097. doi:10.1128/AAC.00486-07 |
| **2** | **66** | Dewi RM. | Angka Kegagalan Pengobatan Klorokuin di Daerah dengan Beda Endemisitasnya; Kajian dengan Teknik PCR dan Kovensional. Badan Litbang Kesehatan RI. 2002. http://repository.bkpk.kemkes.go.id/1607/ |
| **3** | **67** | Estiana L. | Resistensi fansidar dan klorokuin:: Kajian uji sensitifitas serta faktor-faktor yang berhubungan dengan terjadinya resistensi di Kecamatan Pituruh Kabupaten Purworejo [masters thesis]. Yogyakarta: Universitas Gadjah Mada; 2000. |
| **4** | **68** | Sutanto I, Supriyanto S, Ruckert P, Purnomo, Maguire JD, Bangs MJ. | Comparative efficacy of chloroquine and sulfadoxine-pyrimethamine for uncomplicated Plasmodium falciparum malaria and impact on gametocyte carriage rates in the East Nusatenggara province of Indonesia. Am J Trop Med Hyg. 2004;70(5):467-473. |
| **5** | **69** | Arubusman M. | Evaluasi hasil guna kombinasi artesunat-amodiakuin dan primakuin pada pengobatan malaria falciparum tanpa komplikasi di Kabupaten Alor Propinsi Nusa Tenggara Timur [masters thesis]. Yogyakarta: Universitas Gadjah Mada; 2009. |
| **6** | **70** | Triglia T, Wang P, Sims PF, Hyde JE, Cowman AFJTEj | Allelic exchange at the endogenous genomic locus in Plasmodium falciparum proves the role of dihydropteroate synthase in sulfadoxine-resistant malaria. 1998, 17:3807-3815 |
| **7** | **71** | Tjitra E, Suprianto S, Currie BJ, Morris PS, Saunders JR, Anstey NM. | Therapy of uncomplicated falciparum malaria: a randomized trial comparing artesunate plus sulfadoxine-pyrimethamine versus sulfadoxine-pyrimethamine alone in Irian Jaya, Indonesia. Am J Trop Med Hyg. 2001;65(4):309-317. doi:10.4269/ajtmh.2001.65.309 |
| **8** | **12** | Maguire JD, Susanti AI, Krisin, Sismadi P, Fryauff DJ, Baird JK. | The T76 mutation in the pfcrt gene of Plasmodium falciparum and clinical chloroquine resistance phenotypes in Papua, Indonesia. Ann Trop Med Parasitol. 2001;95(6):559-572. doi:10.1080/00034980120092516 |
| **9** | **13** | Nagesha HS, Din-Syafruddin, Casey GJ, et al. | Mutations in the pfmdr1, dhfr and dhps genes of Plasmodium falciparum are associated with in-vivo drug resistance in West Papua, Indonesia. Trans R Soc Trop Med Hyg. 2001;95(1):43-49. doi:10.1016/s0035-9203(01)90329-3 |
| **10** | **44** | Fryauff DJ, Leksana B, Masbar S, et al. | The drug sensitivity and transmission dynamics of human malaria on Nias Island, North Sumatra, Indonesia. Ann Trop Med Parasitol. 2002;96(5):447-462. doi:10.1179/000349802125001249 |
| **11** | **45** | World Health Organization. | World health statistics 2018: monitoring health for the SDGs, sustainable development goals. 2018‎. World Health Organization. https://apps.who.int/iris/handle/10665/272596. |
| **12** | **46** | World Health Organization. | Artemisinin resistance and artemisinin-based combination therapy efficacy: a status report. 2018. World Health Organization. https://apps.who.int/iris/handle/10665/274362. |
| **13** | **47** | Ministry of Health, Republic of Indonesia. | Epidemiologi Malaria di Indonesia. 2011. https://pusdatin.kemkes.go.id/resources/download/pusdatin/buletin/buletin-malaria.pdf |
| **14** | **72** | Asih PBS, Rozi IE, Dewayanti FK, et al. | Efficacy and safety of dihydroartemisinin-piperaquine for the treatment of uncomplicated Plasmodium falciparum and Plasmodium vivax malaria in Papua and Sumatra, Indonesia. Malar J. 2022;21(1):95. Published 2022 Mar 19. doi:10.1186/s12936-022-04101-0 |
| **15** | **73** | Suwanarusk R, Russell B, Chavchich M, et al. | Chloroquine-resistant Plasmodium vivax: in vitro characterization and association with molecular polymorphisms. PLoS One. 2007;2(10):e1089. Published 2007 Oct 31. doi:10.1371/journal.pone.0001089 |
| **16** | **74** | Ratcliff A, Siswantoro H, Kenangalem E, et al. | Therapeutic response of multidrug-resistant Plasmodium falciparum and P. vivax to chloroquine and sulfadoxine-pyrimethamine in southern Papua, Indonesia. Trans R Soc Trop Med Hyg. 2007;101(4):351-359. doi:10.1016/j.trstmh.2006.06.008 |
| **17** | **75** | Poespoprodjo JR, Kenangalem E, Wafom J, et al. | Therapeutic Response to Dihydroartemisinin-Piperaquine for P. falciparum and P. vivax Nine Years after Its Introduction in Southern Papua, Indonesia. Am J Trop Med Hyg. 2018;98(3):677-682. doi:10.4269/ajtmh.17-0662 |
| **18** | **76** | Fryauff DJ, Soekartono, Tuti S, et al. | Survey of resistance in vivo to chloroquine of Plasmodium falciparum and P. vivax in North Sulawesi, Indonesia. Trans R Soc Trop Med Hyg. 1998;92(1):82-83. doi:10.1016/s0035-9203(98)90966-x |
| **19** | **77** | Murphy GS, Basri H, Purnomo, et al. | Vivax malaria resistant to treatment and prophylaxis with chloroquine. Lancet. 1993;341(8837):96-100. doi:10.1016/0140-6736(93)92568-e |
| **20** | **78** | Kim ES, Na BK, Park Y-K, et al. | A case of chloroquine-resistant plasmodium vivax malaria imported from Indonesia. Infection and Chemotherapy. 2008;40(1):52. doi:10.3947/ic.2008.40.1.52 |
| **21** | **79** | Tjitra E, Baker J, Suprianto S, Cheng Q, Anstey NM. | Therapeutic efficacies of artesunate-sulfadoxine-pyrimethamine and chloroquine-sulfadoxine-pyrimethamine in vivax malaria pilot studies: relationship to Plasmodium vivax dhfr mutations. Antimicrob Agents Chemother. 2002;46(12):3947-3953. doi:10.1128/AAC.46.12.3947-3953.2002 |
| **22** | **80** | Salwati E, Handayani S, Jekti RP. | Identifikasi Single Nucleotide Polymorphism (SNP) Gen pvmdr1 pada Penderita Malaria Vivaks di Minahasa Tenggara (Sulawesi Utara). J Biotek Medisiana Indones. 2014;3(2): 49-57 |
| **23** | **81** | Tjitra E, Maladi M, Prasetyorini B, Suprianto S, Harun S, Nurhayati N, Yuwarni E, Yandri B, Laihad F, Ringwald P. | Efficacy of chloroquine, chloroquine plus sulphadoxine-pyrimethamine, and amodiaquine for treatment of vivax malaria in Bangka island, Indonesia: a randomized trial. Med J Indones. 2008;(17): 96-106 |
| **24** | **82** | Asih PB, Rozi IE, Herdiana, et al. | The baseline distribution of malaria in the initial phase of elimination in Sabang Municipality, Aceh Province, Indonesia. Malar J. 2012;11:291. Published 2012 Aug 21. doi:10.1186/1475-2875-11-291 |
| **25** | **83** | Fryauff DJ, Baird JK, Basri H, et al. | Randomised placebo-controlled trial of primaquine for prophylaxis of falciparum and vivax malaria. Lancet. 1995;346(8984):1190-1193. doi:10.1016/s0140-6736(95)92898-7 |
| **26** | **84** | Baird JK, Basri H, Subianto B, et al. | Treatment of chloroquine-resistant Plasmodium vivax with chloroquine and primaquine or halofantrine. J Infect Dis. 1995;171(6):1678-1682. doi:10.1093/infdis/171.6.1678 |
| **27** | **85** | Pasaribu AP, Chokejindachai W, Sirivichayakul C, et al. | A randomized comparison of dihydroartemisinin-piperaquine and artesunate-amodiaquine combined with primaquine for radical treatment of vivax malaria in Sumatera, Indonesia. J Infect Dis. 2013;208(11):1906-1913. doi:10.1093/infdis/jit407 |
| **28** | **86** | Baird JK, Sustriayu Nalim MF, Basri H, et al. | Survey of resistance to chloroquine by Plasmodium vivax in Indonesia. Trans R Soc Trop Med Hyg. 1996;90(4):409-411. doi:10.1016/s0035-9203(96)90526-x |
| **29** | **87** | Baird JK, Wiady I, Fryauff DJ, et al. | In vivo resistance to chloroquine by Plasmodium vivax and Plasmodium falciparum at Nabire, Irian Jaya, Indonesia. Am J Trop Med Hyg. 1997;56(6):627-631. doi:10.4269/ajtmh.1997.56.627 |
| **30** | **11** | Fryauff DJ, Baird JK, Candradikusuma D, et al. | Survey of in vivo sensitivity to chloroquine by Plasmodium falciparum and P. vivax in Lombok, Indonesia. Am J Trop Med Hyg. 1997;56(2):241-244. doi:10.4269/ajtmh.1997.56.241 |
| **31** | **12** | Maguire JD, Susanti AI, Krisin, Sismadi P, Fryauff DJ, Baird JK. | The T76 mutation in the pfcrt gene of Plasmodium falciparum and clinical chloroquine resistance phenotypes in Papua, Indonesia. Ann Trop Med Parasitol. 2001;95(6):559-572. doi:10.1080/00034980120092516 |
| **32** | **44** | Fryauff DJ, Leksana B, Masbar S, et al. | The drug sensitivity and transmission dynamics of human malaria on Nias Island, North Sumatra, Indonesia. Ann Trop Med Parasitol. 2002;96(5):447-462. doi:10.1179/000349802125001249 |
| **33** | **88** | Sutanto I, Suprijanto S, Nurhayati, Manoempil P, Baird JK. | Resistance to chloroquine by Plasmodium vivax at Alor in the Lesser Sundas Archipelago in eastern Indonesia. Am J Trop Med Hyg. 2009;81(2):338-342. |
| **34** | **63** | Asih PBS, Rozi IE, Dewayanti FK, et al. | Efficacy and safety of dihydroartemisinin-piperaquine for the treatment of uncomplicated plasmodium falciparum and plasmodium vivax malaria in northern Papua and Jambi, Indonesia. medRxiv. 2020. doi:10.1101/2020.09.04.20188706 |
| **35** | **15** | Syafruddin D, Asih PB, Aggarwal SL, Shankar AH. | Frequency distribution of antimalarial drug-resistant alleles among isolates of Plasmodium falciparum in Purworejo district, Central Java Province, Indonesia. Am J Trop Med Hyg. 2003;69(6):614-620. |
| **36** | **24** | Huaman MC, Yoshinaga K, Suryanatha A, Suarsana N, Kanbara H. | Short report: polymorphisms in the chloroquine resistance transporter gene in Plasmodium falciparum isolates from Lombok, Indonesia. Am J Trop Med Hyg. 2004;71(1):40-42. |
| **37** | **30** | Syafruddin D, Asih PB, Wahid I, et al. | Malaria prevalence in Nias District, North Sumatra Province, Indonesia. Malar J. 2007;6:116. Published 2007 Aug 30. doi:10.1186/1475-2875-6-116 |
| **38** | **116** | Syafruddin D, Asih PB, Casey GJ, et al. | Molecular epidemiology of Plasmodium falciparum resistance to antimalarial drugs in Indonesia. Am J Trop Med Hyg. 2005;72(2):174-181. |
| **39** | **9** | Syafruddin D, Krisin, Asih P, et al. | Seasonal prevalence of malaria in West Sumba district, Indonesia. Malar J. 2009;8:8. Published 2009 Jan 9. doi:10.1186/1475-2875-8-8 |
| **40** | **170** | Kamelia M, Supargiyono, Wijayanti MA. | Study on Chloroquine Resistance Transporter (pfcrt) Gene Polymorphism of Plasmodium falciparum in Malaria Patients in Lampung. Tropical Medicine Jurnal. 2011;1(1). https://doi.org/10.22146/tmj.4567 |
| **41** | **118** | Ali M, Hidayatullah TA, Alimuddin Z, Sabrina Y. | Sequence Diversity of pfmdr1 and Sequence Conserve of pldh in Plasmodium falciparum from Indonesia: Its implications on Designing a Novel Antimalarial Drug with Less Prone to Resistance. Iran J Parasitol. 2013;8(4):522-529. |
| **42** | **57** | Suwandi JF, Asmara W, Kusnanto H, Syafruddin D, Supargiyono S. | Efficacy of Artemisinin Base Combination Therapy and Genetic Diversity of Plasmodium falciparum from Uncomplicated Malaria Falciparum Patient in District of Pesawaran, Province of Lampung, Indonesia. Iran J Parasitol. 2019;14(1):143-150. |
| **43** | **32** | Fitriah F, Sulistyawati S, Riyanto S, et al. | Polymorphism of Plasmodium falciparum dihydrofolate reductase and dihydropteroate synthase genes among pregnant women with falciparum malaria in Banjar District, South Kalimantan Province, Indonesia. Journal of Tropical Life Science. 2012;2(3):92-98. doi:10.11594/jtls.02.03.07 |
| **44** | **132** | Basuki S, Fitriah, Riyanto S, Budiono, Dachlan YP, Uemura H. | Two novel mutations of pfdhps K540T and I588F, affecting sulphadoxine-pyrimethamine-resistant response in uncomplicated falciparum malaria at Banjar district, South Kalimantan Province, Indonesia. Malar J. 2014;13: 135–143. https://doi.org/10.1186/1475-2875-13-135 |
| **45** | **34** | Mukh S, Darlina, Siti N. | DETEKSI SPESIES PARASIT MALARIA BERBASIS 18S RRNA DAN UJI RESISTENSINYA TERHADAP OBAT UNTUK GEN DHPS SEBAGAI PENDUKUNG PENGEMBANGAN VAKSIN MALARIA IRADIASI. Prosiding Seminar PSTA dan UNS: PSTA BATAN 2016:7-13. |
| **46** | **29** | Basuki S, Fitriah, Riyanto S, et al. | Two novel mutations of pfdhps K540T and I588F, affecting sulphadoxine-pyrimethamine-resistant response in uncomplicated falciparum malaria at Banjar district, South Kalimantan Province, Indonesia. Malar J. 2014;13(1): 1-8. https://doi.org/10.1186/1475-2875-13-135 2014;13(1):1-8. |
| **47** | **25** | Saleh I, Handayani D, Anwar C. | Polymorphisms in the pfcrt and pfmdr1 genes in Plasmodium falciparum &gt; isolates from South Sumatera, Indonesia. Med J Indones. 2014;23(1):3-8. https://doi.org/10.13181/mji.v23i1.679 |
| **48** | **171** | Syafruddin. | Efficacy and safety of dihydroartemisinin-piperaquine for the treatment of uncomplicated Plasmodium falciparum and Plasmodium vivax malaria in 4 sentinel sites in Indonesia. 2016. |
| **49** | **172** | Reteng P, Vrisca V, Sukarno I, et al. | Genetic polymorphisms in Plasmodium falciparum chloroquine resistance genes, pfcrt and pfmdr1, in North Sulawesi, Indonesia. BMC Res Notes. 2017;10(1):147. Published 2017 Apr 4. doi:10.1186/s13104-017-2468-1 |
| **50** | **35** | Poespoprodjo JR, Kenangalem E, Wafom J, et al. | Therapeutic Response to Dihydroartemisinin-Piperaquine for P. falciparum and P. vivax Nine Years after Its Introduction in Southern Papua, Indonesia. Am J Trop Med Hyg. 2018;98(3):677-682. doi:10.4269/ajtmh.17-0662 |
| **51** | **75** | Poespoprodjo JR, Kenangalem E, Wafom J, et al. | Therapeutic Response to Dihydroartemisinin-Piperaquine for P. falciparum and P. vivax Nine Years after Its Introduction in Southern Papua, Indonesia. Am J Trop Med Hyg. 2018;98(3):677-682. doi:10.4269/ajtmh.17-0662 |
| **52** | **104** | Lubis IND, Wijaya H, Lubis M, Lubis CP, Beshir KB, Sutherland CJ. | Plasmodium falciparum Isolates Carrying pfk13 Polymorphisms Harbor the SVMNT Allele of pfcrt in Northwestern Indonesia. Antimicrob Agents Chemother. 2020;64(8):e02539-19. Published 2020 Jul 22. doi:10.1128/AAC.02539-19 |
| **53** | **92** | Rachmad B. | Isolasi Dan Identifikasi Mutasi Gen Pfk13 (Pf3d7_1343700) Sebagai Penanda Resistensi Artemisinin Pada Isolat Plasmodium Falciparum Asal Lampung. Prosiding dalam rangka Rakernas XIV & Temu Ilmiah XXII. 2019. 25–40 |
| **54** | **173** | Marfurt J, Wirjanata G, Prayoga P, et al. | Longitudinal ex vivo and molecular trends of chloroquine and piperaquine activity against Plasmodium falciparum and P. vivax before and after the introduction of artemisinin-based combination therapy in Papua, Indonesia. Int J Parasitol Drugs Drug Resist. 2021;17:46-56. doi:10.1016/j.ijpddr.2021.06.002 |
| **55** | **174** | Lamaka B, Arsin A, Alam G, et al. | Plasmodium Falciparum Gene Polymorphisms Pfmdr1 N86Y and Drug Self-medication in the Endemic Areas of West Papua Region, Indonesia. International Journal of Sciences: Basic and Applied Research. 2017;33: 103-111. |
| **56** | **119** | Asih PB, Syafruddin D, Leake J, et al. | Phenotyping clinical resistance to chloroquine in Plasmodium vivax in northeastern Papua, Indonesia. Int J Parasitol Drugs Drug Resist. 2011;1(1):28-32. Published 2011 Oct 5. doi:10.1016/j.ijpddr.2011.08.001 |
| **57** | **36** | Hastings MD, Maguire JD, Bangs MJ, et al. | Novel Plasmodium vivax dhfr alleles from the Indonesian Archipelago and Papua New Guinea: association with pyrimethamine resistance determined by a Saccharomyces cerevisiae expression system. Antimicrob Agents Chemother. 2005;49(2):733-740. doi:10.1128/AAC.49.2.733-740.2005 |
| **58** | **175** | Brega S, de Monbrison F, Severini C, et al. | Real-time PCR for dihydrofolate reductase gene single-nucleotide polymorphisms in Plasmodium vivax isolates. Antimicrob Agents Chemother. 2004;48(7):2581-2587. doi:10.1128/AAC.48.7.2581-2587.2004 |
| **59** | **176** | Suwanarusk R, Chavchich M, Russell B, et al. | Amplification of pvmdr1 associated with multidrug-resistant Plasmodium vivax. J Infect Dis. 2008;198(10):1558-1564. doi:10.1086/592451 |
| **60** | **177** | Kim ES, Na BK, Park Y-K, et al. | A case of chloroquine-resistant plasmodium vivax malaria imported from Indonesia. Infection and Chemotherapy. 2008;40(1):52. doi:10.3947/ic.2008.40.1.52 |
| **61** | **178** | Asih PB, Marantina SS, Nababan R, et al. | Distribution of Plasmodium vivax pvdhfr and pvdhps alleles and their association with sulfadoxine-pyrimethamine treatment outcomes in Indonesia. Malar J. 2015;14:365. Published 2015 Sep 22. doi:10.1186/s12936-015-0903-0 |
